# TSPO and LRBA Expression Is Associated with Acute Renal Allograft Rejection: An Integrative Transcriptomic Analysis

**DOI:** 10.3390/ijms27219830

**Published:** 2026-11-02

**Authors:** Sichuan Yi, Qiucheng Zhu, Song Chen

**Affiliations:** 1 Institute of Organ Transplantation, Tongji Hospital, Tongji Medical College, Huazhong University of Science and Technology; Key Laboratory of Organ Transplantation, Ministry of Education; NHC Key Laboratory of Organ Transplantation; Key Laboratory of Organ Transplantation, Chinese Academy of Medical Sciences; Hubei Provincial Clinical Research Center for Organ Transplantation; Wuhan 430030, China; 2 Li-Yuan Hospital, Tongji Medical College, Huazhong University of Science and Technology, Wuhan 430077, China; 3 Laboratory of Metabolic Abnormalities and Vascular Aging, Huazhong University of Science and Technology, Wuhan 430077, China

**Keywords:** macrophages, mitophagy, machine learning, single-cell RNA sequencing, biomarkers, kidney transplantation, acute rejection

## Abstract

Acute renal transplant rejection causes allograft injury and affects long-term graft survival. Mitophagy helps maintain mitochondrial homeostasis and regulate immune function, but its relationship with acute rejection remains unclear. We integrated public renal allograft transcriptomic datasets using differential expression analysis, weighted gene co-expression network analysis, and machine learning. We performed external cohort validation, survival analysis, immune deconvolution, single-cell RNA sequencing analysis, virtual knockout analysis, and quantitative PCR in mouse renal allografts. Among 32 shared mitophagy-associated genes which we identified, TSPO was consistently upregulated and LRBA was downregulated across independent biopsy cohorts. An exploratory TSPO/LRBA two-gene model showed modest discrimination between rejection and non-rejection samples. Higher model scores were associated with poorer graft survival. An exploratory analysis of the model was then performed in peripheral blood. Functional and single-cell analyses linked these genes to monocyte/macrophage populations, antigen presentation, T-cell signaling, and inflammatory pathways. Virtual knockout analysis predicted changes in antigen presentation, phagocytosis, and allograft rejection pathways. Quantitative PCR confirmed increased TSPO and decreased LRBA expression in mouse renal allografts with acute rejection. Together, these findings identify TSPO and LRBA as new candidate mitophagy-associated biomarkers linked to monocytes/macrophages and support further investigation of their associations with acute renal transplant rejection.

## 1. Introduction

For end-stage renal disease (ESRD), kidney transplantation is the treatment of choice, but acute rejection (AR) remains an important challenge to achieving favorable graft outcomes. AR may occur anytime after transplantation [1,2] and remains a significant cause of long-term graft dysfunction and reduced graft survival [3,4,5]. AR results from antigen-specific T-cell responses and/or antibody responses against the graft and is mainly classified as T-cell-mediated rejection (TCMR) or antibody-mediated rejection (ABMR) [6,7,8]. Although immunosuppressive treatment has lowered the rate of AR during the first post-transplant year [9,10,11,12], long-term graft survival remains unsatisfactory [13].

Serum creatinine (Scr), glomerular filtration rate (GFR), urine output, and renal biopsy are commonly used to assess AR in clinical practice [14,15]. Renal biopsy is the most reliable method for detecting subclinical rejection, but its invasiveness, cost, and sampling limitations make frequent monitoring difficult [16,17]. More sensitive and specific biomarkers are therefore needed to help identify renal allograft rejection earlier.

Macrophages play a key role in renal allograft inflammation. Their infiltration and activation have been associated with acute rejection after kidney transplantation [16,18]. Macrophage functional states span a spectrum, with M1-like cells promoting inflammation and M2-like cells helping to limit it. M1-like macrophages release inflammatory mediators that can worsen renal tissue injury, whereas M2-like macrophages are generally associated with tissue repair and graft protection [19,20]. Metabolism and organelles affect macrophage function. Mitophagy selectively removes and recycles damaged mitochondria, helping to maintain mitochondrial balance, support cell survival, and regulate macrophage function [21,22,23,24,25].

Mitophagy has been linked to kidney allograft rejection. A previous study reported downregulation of the mitophagy pathway and reduced expression of the mitophagy-related genes PINK1 and PRKN in human kidney allograft biopsies with acute T-cell-mediated rejection [26]. Meanwhile, a study reported that loss of the mitophagy-related genes (PINK1 or PRKN) promoted macrophage polarization from M0 to an M2-like phenotype in experimental and human kidney fibrosis [27].

Noninvasive methods are increasingly being developed to monitor kidney allograft rejection. These methods use blood biomarkers, urine biomarkers, or a combination of tests. Blood biomarkers include donor-specific antibodies (DSAs), donor-derived cell-free DNA (dd-cfDNA), and gene-expression classifiers [28]. DSAs indicate an antibody response against the donor and help assess antibody-mediated rejection [28,29]. dd-cfDNA reflects allograft injury [30]. Blood gene expression classifiers detect immune signatures associated with rejection [31]. Urine biomarkers, such as CXCL9 and CXCL10, reflect inflammation in the graft. However, infections can also increase their levels [32]. Combining these tests may provide additional information [33]. Their diagnostic performance and clinical utility still need validation in specific clinical settings [28].

Previous studies have linked mitophagy to kidney allograft rejection. However, the expression of mitophagy-related genes in macrophages and their potential value as rejection markers remain unclear. We aimed to identify these genes and assess their association with acute rejection. We analyzed public bulk and single-cell transcriptomic datasets (Supplementary Table S1). We combined differential expression analysis, weighted gene co-expression network analysis, and a mitophagy-related gene set to identify candidate genes. We then examined their expression in macrophages using single-cell data. We also built an exploratory two-gene model and assessed its association with rejection status and graft outcomes. These analyses may help identify candidate markers and guide further studies of macrophage-associated processes in kidney allograft rejection.

## 2. Results

### 2.1. Identification of Mitophagy-Associated Hub Genes Associated with Kidney Transplant Rejection

**Figure 1:**
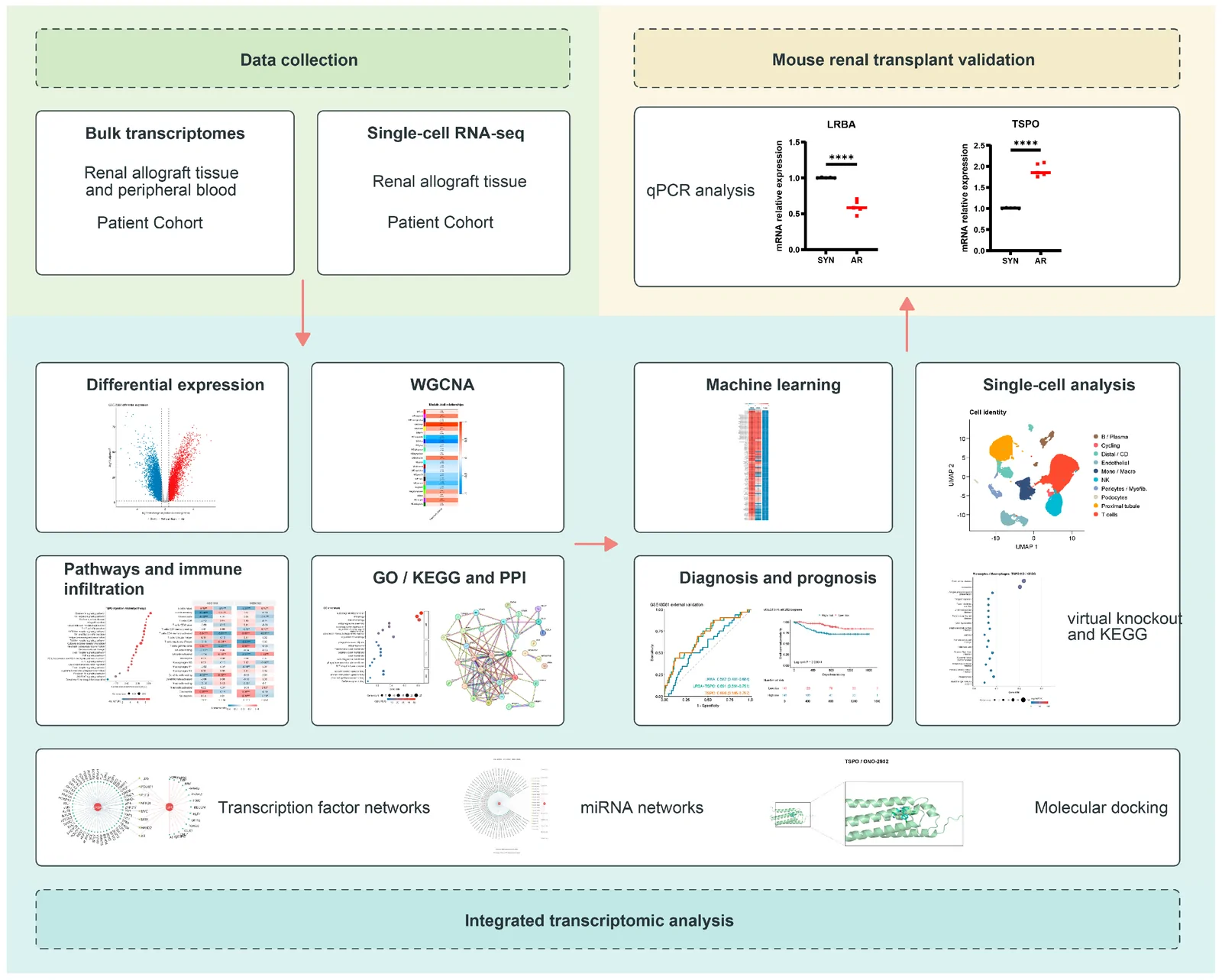
Study workflow. Overview of data collection, differential expression analysis, WGCNA, machine learning screening, diagnostic and prognostic evaluation, pathway and immune infiltration analyses, single-cell analysis and virtual knockout, regulatory network analysis, molecular docking, and validation in mouse kidney transplantation model.

Figure 1 shows the study workflow. We first downloaded GSE25902, a dataset of human renal allograft rejection after transplantation. After examining the samples, we included 120 samples, consisting of 96 non-rejection and 24 acute rejection samples.

**Figure 2:**
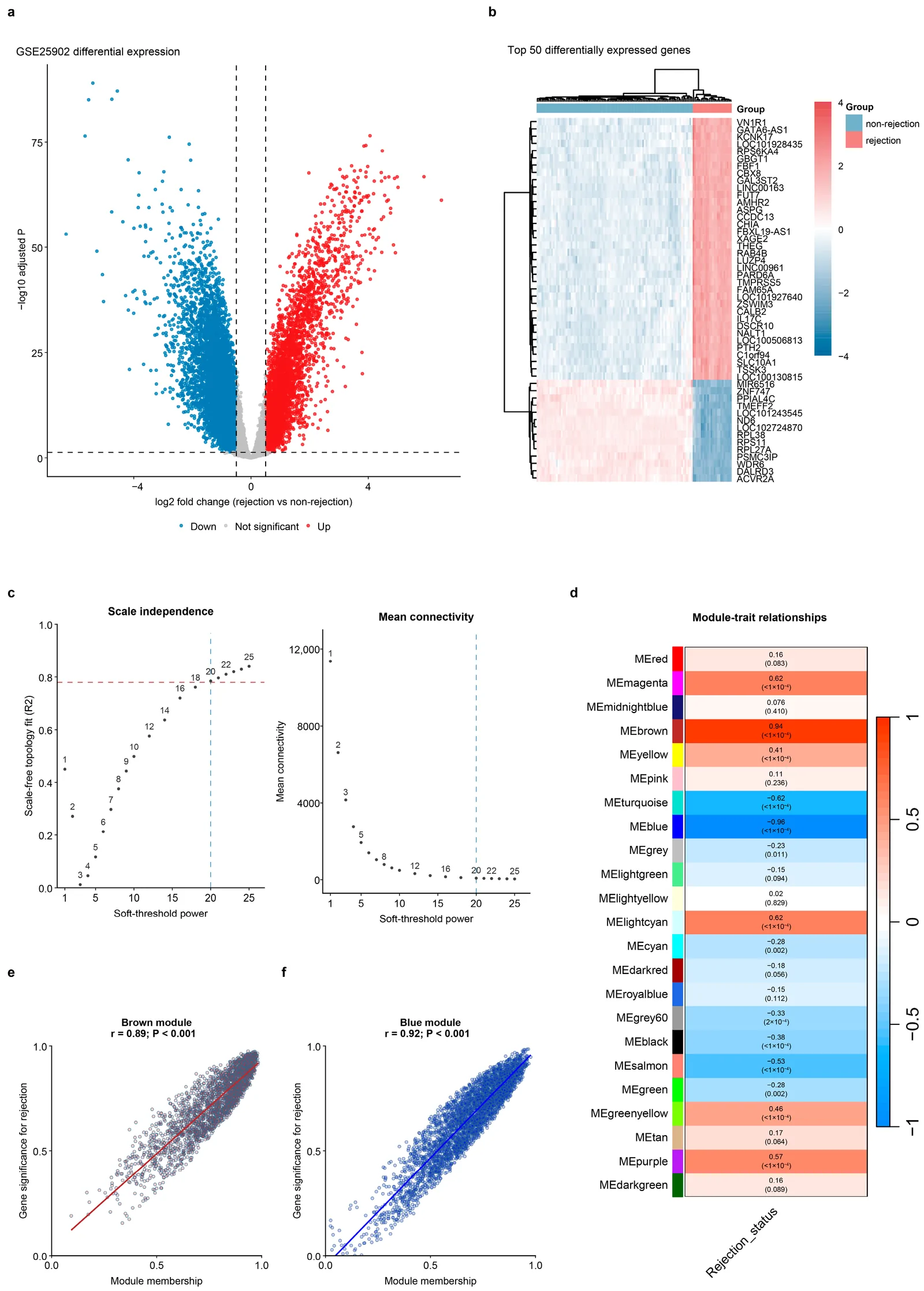
Identification of mitophagy-associated hub genes associated with kidney transplant rejection. (**a**) Volcano plot of expression differences between rejection and non-rejection samples in GSE25902. (**b**) Expression patterns of top 50 differentially expressed genes displayed as heatmap. (**c**) Selection of WGCNA soft-thresholding power based on scale-free topology fit index and mean connectivity. (**d**) Module eigengene associations with rejection status presented as heatmap. (**e**,**f**) Relationships between absolute module membership and gene significance in brown and blue modules.

We performed differential expression analysis to identify genes associated with kidney transplant rejection. We identified 11,395 differentially expressed genes with the threshold (|log2FC| > 0.5 and adjusted *p* < 0.05). These included 4410 upregulated genes and 6985 downregulated genes. Volcano plots and heatmaps showed the overall expression distribution of these genes and a clear transcriptional difference between rejection and non-rejection samples (Figure 2a,b). Sensitivity analyses showed that the directions of the TSPO and LRBA expression differences were consistent across changes in sample inclusion, normalization and adjustment for available covariates (Supplementary Figure S1). We next used WGCNA to find gene modules associated with rejection. Based on the scale-free topology fit and mean connectivity, we chose 20 as the soft-thresholding power for the adjacency matrix. This was the lowest power at which the fit index reached 0.80 (Figure 2c). The brown and blue modules had the strongest associations with rejection status (Figure 2d), and both were strongly correlated with renal allograft rejection (Figure 2e,f).

### 2.2. Functional Analysis and Protein Interaction Networks

**Figure 3:**
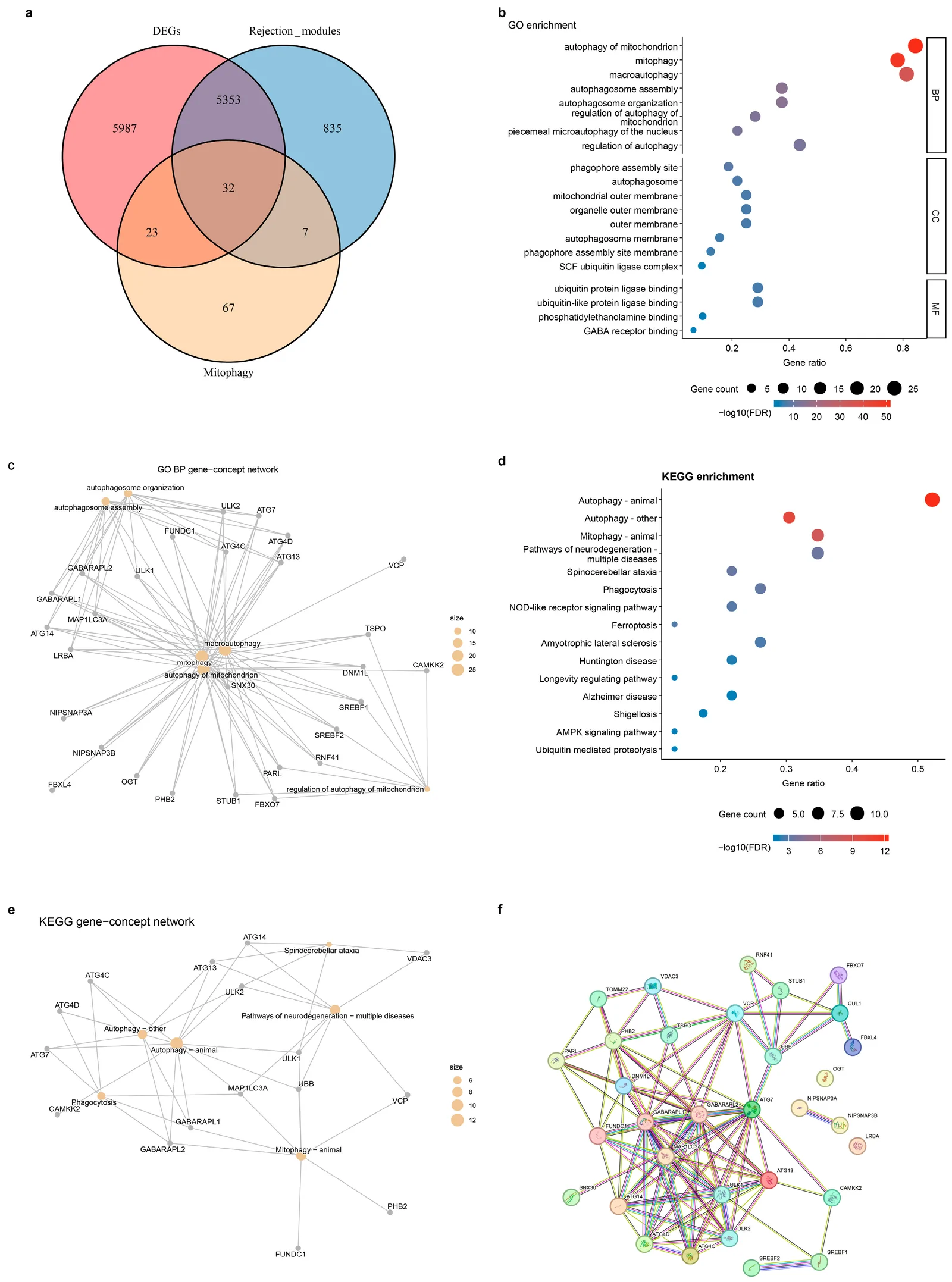
Functional analysis and protein interaction networks. (**a**) Overlap of differential expression results, rejection-associated WGCNA modules, and mitophagy gene set, displayed in Venn diagram. (**b**) Enrichment results for overlapping genes based on Gene Ontology (GO). (**c**) Gene–concept network linking these genes to significantly enriched GO terms. (**d**) Enrichment results based on Kyoto Encyclopedia of Genes and Genomes (KEGG) pathways. (**e**) Gene–concept network connecting overlapping genes with significantly enriched KEGG pathways. (**f**) Protein–protein interaction (PPI) network for overlapping genes.

We used genes from the blue and brown modules for further analysis because these modules showed the strongest correlations with rejection. Comparing the differential expression results, WGCNA module genes, and mitophagy gene set yielded 32 candidate hub genes in renal rejection, as shown in the Venn diagram (Figure 3a). GO analysis linked these genes mainly to biological processes involving mitophagy and autophagy, autophagosome-associated cellular structures, and ubiquitin-dependent regulatory functions (Figure 3b). The gene–concept network showed the candidate genes associated with the enriched GO terms and the genes shared across these terms (Figure 3c). KEGG analysis showed enrichment of autophagy and mitochondrial quality control related pathways, including autophagy, mitophagy, phagocytosis, and ubiquitin-mediated proteolysis. Additional enriched pathways included NOD-like receptor signaling and ferroptosis, which involve immune/inflammatory responses and cell death. Together, these findings point to possible roles for the hub genes in mitochondrial autophagy, activation of immune and inflammatory responses, and stress-related cell death during kidney transplant rejection (Figure 3d). The KEGG gene–concept network showed the candidate genes associated with the enriched pathways and their overlap across pathways (Figure 3e). PPI network analysis showed potential functional interactions among the hub genes (Figure 3f). This suggests that these genes may act together in immune regulatory and mitochondrial pathways associated with rejection.

### 2.3. Machine Learning Screening Prioritized TSPO and LRBA as Key Candidate Genes

**Figure 4:**
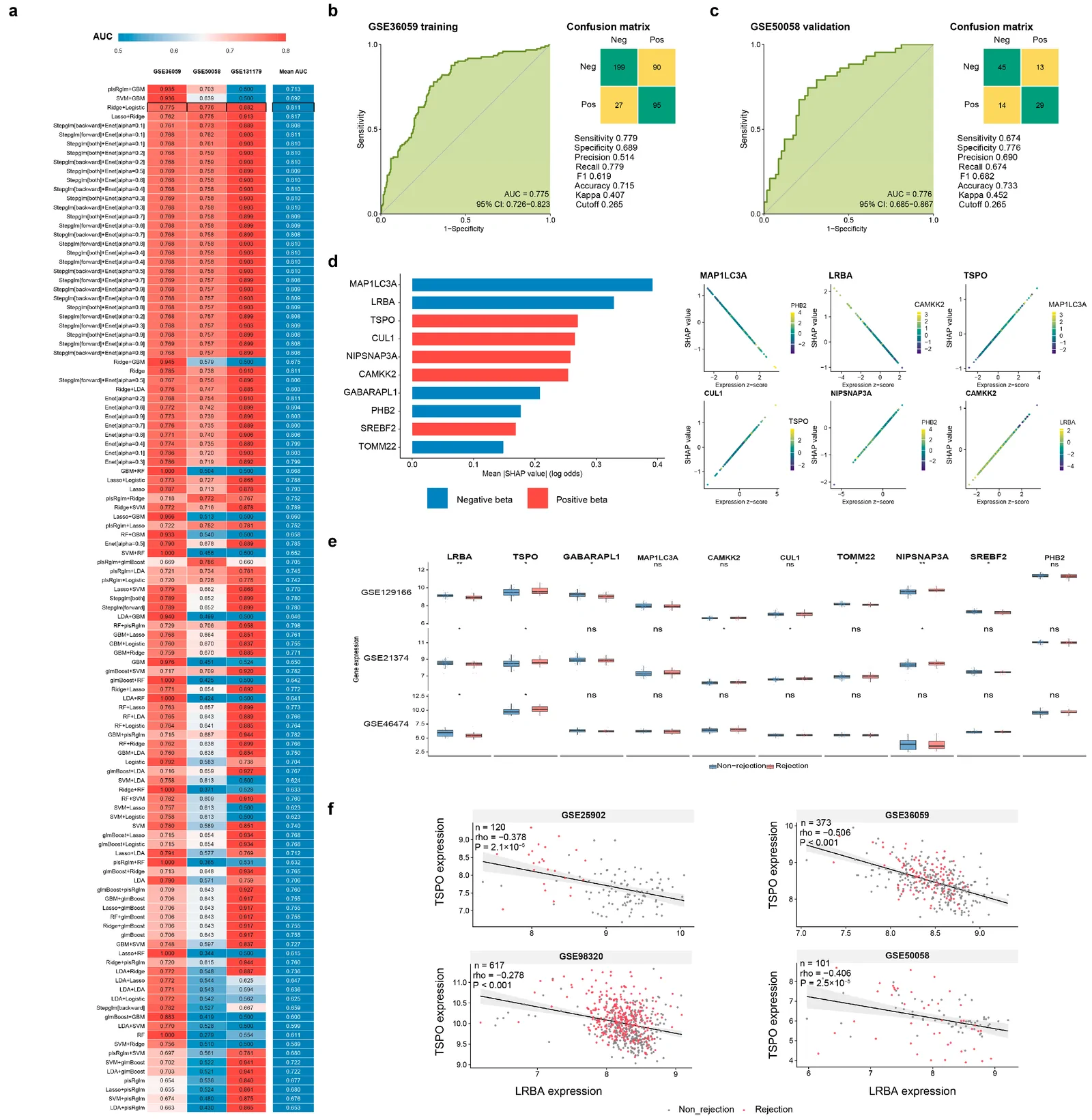
Machine learning screening prioritized TSPO and LRBA as key candidate genes. (**a**) Comparison of 113 machine learning combinations based on receiver operating characteristic (ROC) curves and their area under the curve (AUC). The highlighted combination is Ridge + Logistic. (**b**,**c**) ROC plots, confusion matrices, and classification metrics for GSE36059 (training) and GSE50058 (validation), respectively. (**d**) Mean absolute SHAP values for the ten Ridge + Logistic model genes and SHAP dependence plots for the six leading genes. (**e**) Expression of the ten model genes in rejection and non-rejection samples from GSE129166, GSE21374 and GSE46474. Group differences were tested with the two-sided Wilcoxon rank-sum test. * *p* < 0.05; ** *p* < 0.01. ns, not significant. (**f**) The relationships between LRBA and TSPO expression in the four cohorts.

We trained 113 combinations of machine learning algorithms on GSE36059 and evaluated them in GSE50058. The Ridge + Logistic model was selected for final presentation and further analyses (Figure 4a). Ridge + Logistic achieved an AUC of 0.882 (95% CI, 0.770–0.994) in test cohort, suggesting good generalizability. (Figure 4a).

We further evaluated the Ridge + Logistic model using ROC curves and confusion matrix analysis in the training and validation cohorts. In the GSE36059 training cohort, the model achieved an AUC of 0.775 (95% CI: 0.726–0.823), with an accuracy of 0.715, sensitivity of 0.779, specificity of 0.689, and F1 score of 0.619 (Figure 4b). In the GSE50058 validation cohort, the model showed comparable performance, with an AUC of 0.776 (95% CI: 0.685–0.867), accuracy of 0.733, sensitivity of 0.674, specificity of 0.776, and F1 score of 0.682 (Figure 4c). The model contained 10 genes: MAP1LC3A, LRBA, TSPO, CUL1, NIPSNAP3A, CAMKK2, GABARAPL1, PHB2, SREBF2, and TOMM22. We performed SHAP analysis in the GSE36059 training cohort to examine the contribution of each gene to the model (Figure 4d). Ranking by mean absolute SHAP values showed that MAP1LC3A, LRBA, TSPO, CUL1, NIPSNAP3A, and CAMKK2 were the top contributors to model prediction. MAP1LC3A and LRBA had negative regression coefficients, whereas TSPO, CUL1, NIPSNAP3A, and CAMKK2 had positive coefficients. These coefficients indicated opposite directions of contribution to the predicted rejection risk. SHAP dependence plots further showed gene-specific relationships between standardized expression levels and model output.

We next examined the expression of the 10 model genes across multiple external datasets. LRBA expression was consistently lower and TSPO expression was consistently higher in rejection samples than in non-rejection samples across both renal allograft biopsy and peripheral blood datasets. (Figure 4e). TSPO and LRBA expression showed negative correlations across the four cohorts shown in Figure 4f.

These results indicate that LRBA and TSPO showed opposite but reproducible expression patterns during renal allograft rejection.

### 2.4. Discrimination of Rejection by TSPO, LRBA and the Two-Gene Model

To explore the ability of combined LRBA and TSPO expression to distinguish rejection from non-rejection samples, we constructed a two-gene logistic regression model using GSE98320 as the training cohort. LRBA and TSPO expression values were standardized by subtracting their respective training means and dividing by their training standard deviations before entering the model. The final model was defined as follows: logit (*p*) = 0.259492 − 0.103523 × z_LRBA + 0.662075 × z_TSPO, where *p* represents the predicted probability of rejection.

**Figure 5:**
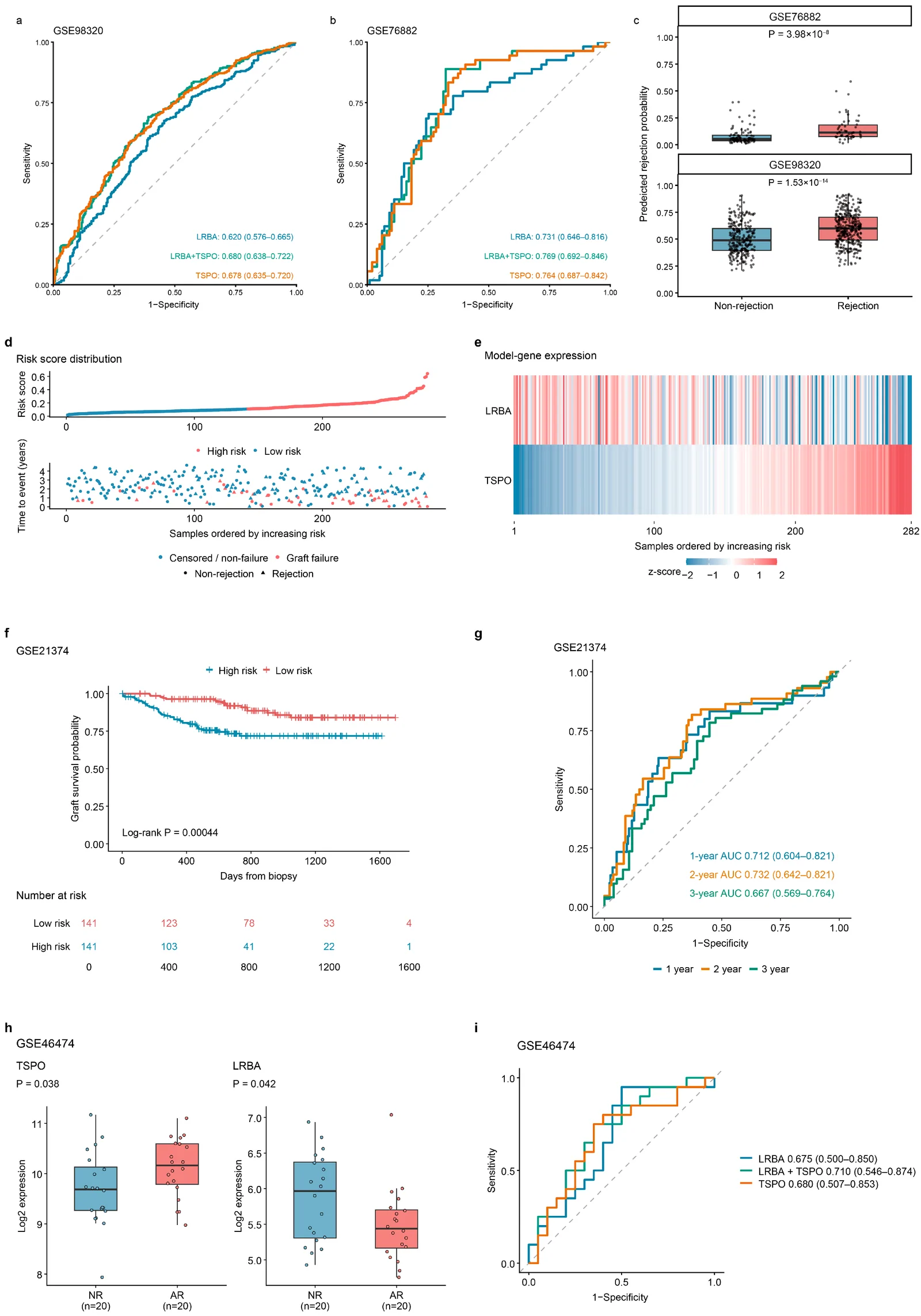
Discrimination of rejection by TSPO, LRBA and the two-gene model. (**a**,**b**) ROC curves for TSPO, LRBA, and two-gene model using GSE98320 for training and GSE76882 for validation, respectively. (**c**) Predicted rejection probabilities for rejection and non-rejection samples in both cohorts. (**d**) Risk scores and corresponding graft outcomes in GSE21374. (**e**) Heatmap of LRBA and TSPO expression, with samples arranged from lowest to highest risk score. (**f**) Graft survival in two risk groups, estimated by Kaplan–Meier method, with numbers at risk displayed. (**g**) ROC curves for graft outcomes at 1, 2, and 3 years. (**h**) TSPO and LRBA expression in peripheral blood samples from acute rejection and non-rejection groups in GSE46474. (**i**) ROC curves for TSPO, LRBA, and two-gene model in GSE46474.

The GSE98320 training cohort contained 343 samples with rejection and 274 without rejection. The LRBA/TSPO model yielded an AUC of 0.680 (Figure 5a). In GSE76882, which included 54 rejection and 99 non-rejection samples, the model yielded an AUC of 0.769 (Figure 5b). In both cohorts, model-predicted probabilities were significantly greater for samples with rejection than for those without rejection (Figure 5c). These results indicate modest discrimination between rejection and non-rejection samples. Calibration plots are provided in Supplementary Figure S2.

We also evaluated TSPO and LRBA individually and assessed whether their combination improved discrimination. TSPO alone yielded AUCs of 0.678 in GSE98320 and 0.764 in GSE76882. The corresponding AUCs for the two-gene model were 0.680 and 0.769. Adding LRBA did not significantly improve the AUC in either cohort (paired DeLong *p* = 0.514 and 0.372, respectively). These results did not demonstrate additional discrimination from combining LRBA with TSPO. We then assessed the prognostic performance of the LRBA/TSPO model in GSE21374. Using the median risk score as the cutoff, we assigned patients to two risk groups. The scores showed a clear separation between these groups (Figure 5d). Consistent with the model coefficients, patients with higher scores showed increased TSPO expression but decreased LRBA expression (Figure 5e). Kaplan–Meier curves indicated significantly worse long-term graft survival among patients with higher risk scores (Figure 5f). Time-dependent ROC analysis also indicated moderate prognostic performance. The AUC was 0.712 at one year, 0.732 at two years, and 0.667 at three years (Figure 5g).

To examine the potential use of the TSPO/LRBA signature as a minimally invasive marker, we further explored the expression pattern and model discrimination in GSE46474. TSPO expression was higher and LRBA expression was lower in blood samples from the rejection group, consistent with the expression directions in graft tissue (Figure 5h). Receiver operating characteristic (ROC) analysis further showed moderate discriminatory potential of the two-gene signature in peripheral blood (Figure 5i). These preliminary blood findings warrant further evaluation in larger independent cohorts. The peripheral blood findings provide preliminary evidence of a similar expression pattern outside renal biopsy samples.

### 2.5. TSPO/LRBA Expression States Are Associated with Immune Activation in Rejection

**Figure 6:**
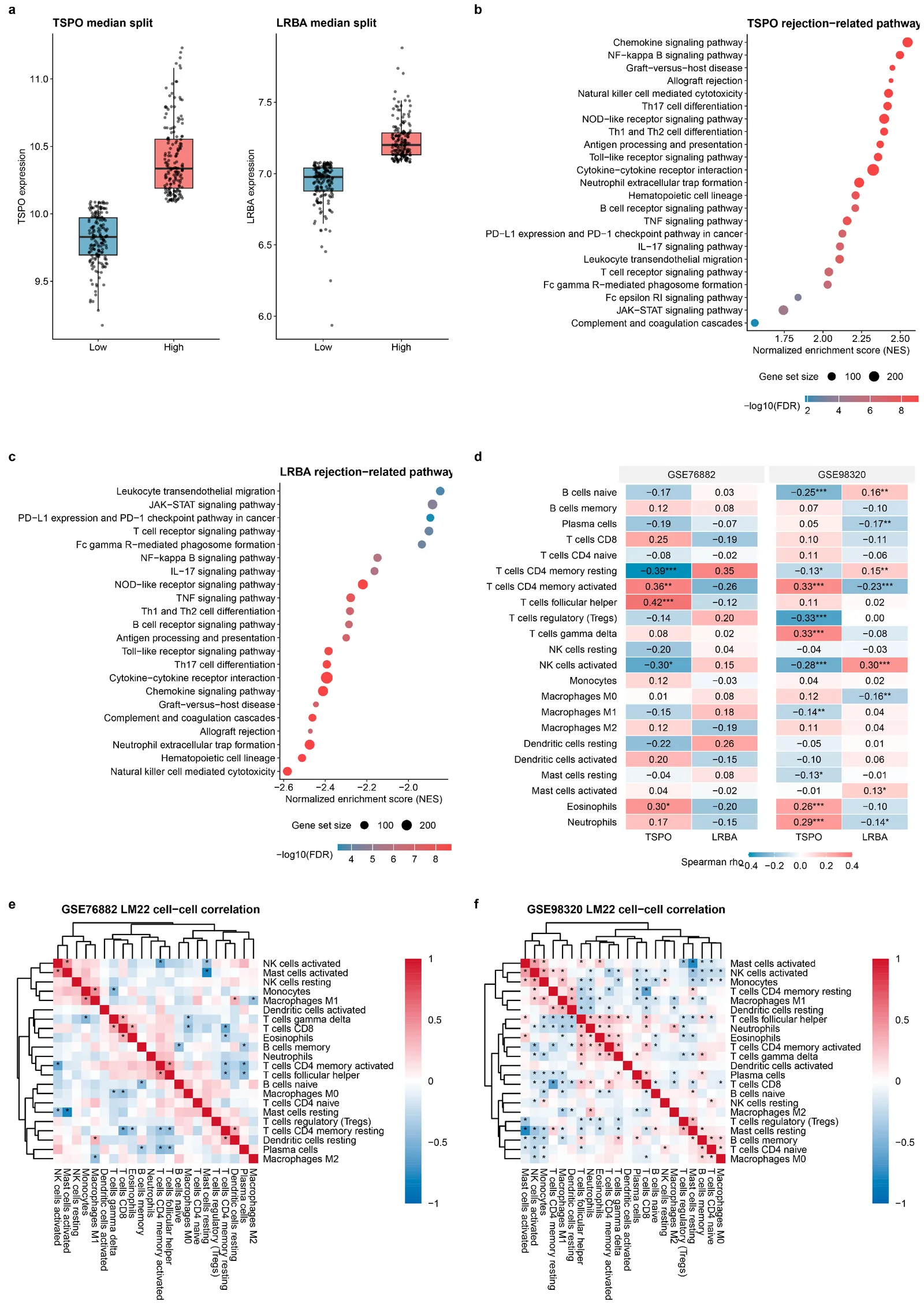
TSPO/LRBA expression states are associated with immune activation in rejection. (**a**) TSPO and LRBA expression in high- and low-expression groups defined by median expression of each gene among rejection samples in GSE98320. (**b**,**c**) Gene set enrichment analysis of rejection-related KEGG pathways associated with TSPO and LRBA expression, respectively. (**d**) Heatmap of correlations between TSPO/LRBA expression and immune cell fractions estimated by CIBERSORT in GSE76882 and GSE98320. (**e**,**f**) Correlation matrices for 22 immune cell fractions in GSE76882 and GSE98320, respectively. * adjusted *p* < 0.05; ** adjusted *p* < 0.01; *** adjusted *p* < 0.001.

To examine the biological pathways associated with TSPO and LRBA expression within rejection, we used the median expression of each gene to define high and low expression groups among the 343 rejection samples in GSE98320 (Figure 6a). Enrichment in TSPO-high samples corresponded to positive normalized enrichment scores, while enrichment in LRBA-low samples corresponded to negative scores. In TSPO-high samples, significant enrichment involved several immune and inflammatory pathways associated with rejection. These included chemokine signaling, allograft rejection, graft-versus-host disease, and cytokine–cytokine receptor interaction. Enrichment also involved signaling through NF-kappa B, TNF, IL-17, T cell receptors, NOD-like receptors, and Toll-like receptors, together with Th1/Th2 and Th17 cell differentiation, antigen processing and presentation, natural killer cell-mediated cytotoxicity, complement and coagulation cascades, and Fc gamma R-mediated phagocytosis (Figure 6b).

LRBA-low samples showed enrichment of a largely overlapping set of rejection-related pathways. These included antigen processing and presentation, allograft rejection, graft-versus-host disease, and T-cell receptor signaling. Other enriched pathways involved cytokine and chemokine signaling; signaling through Toll-like receptors, NOD-like receptors, TNF, NF-kappa B, and IL-17; complement and coagulation cascades; and natural killer cell-mediated cytotoxicity (Figure 6c). These findings showed that higher TSPO and lower LRBA expression were associated with immune and inflammatory pathway enrichment within rejection samples. We examined the relationships of TSPO and LRBA expression with immune cell proportions estimated by CIBERSORT. Across two independent datasets, higher TSPO expression was associated with greater proportions of several immune cell types, most notably gamma delta T cells and activated CD4 memory T cells. Conversely, higher LRBA expression was associated with lower proportions of several cell types, particularly activated CD4 memory T cells, gamma delta T cells, and M0 macrophages. These findings showed associations between TSPO/LRBA expression and estimated relative immune cell fractions (Figure 6d). Correlations among these fractions were also observed (Figure 6e,f).

### 2.6. Single-Cell Analysis Characterized TSPO and LRBA Expression Across Kidney Allograft Cell Populations

**Figure 7:**
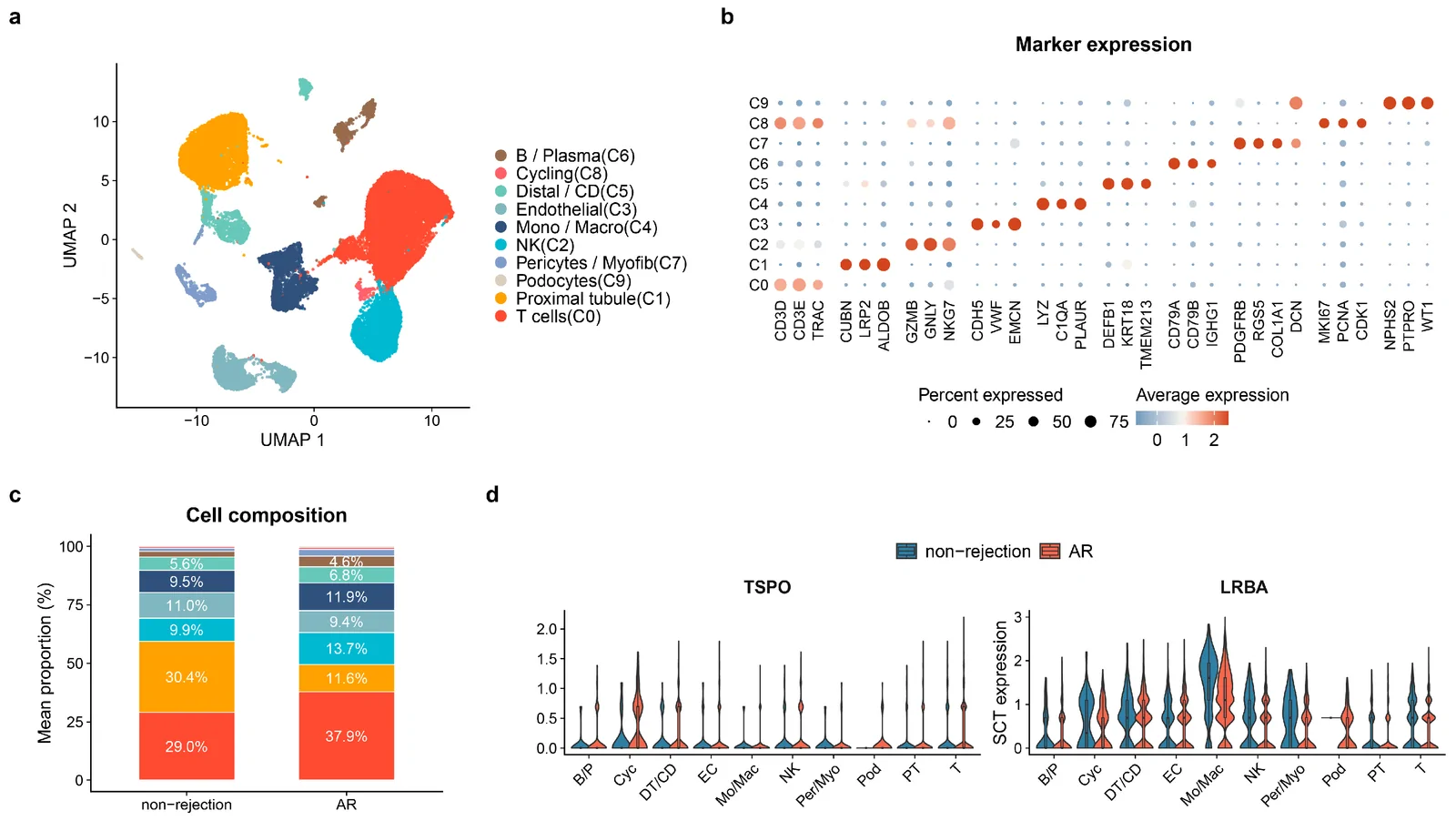
Single-cell analysis characterized TSPO and LRBA expression across kidney allograft cell populations. (**a**) Ten cell types identified in GSE189536, visualized by uniform manifold approximation and projection (UMAP). (**b**) Representative markers for cell type annotation displayed in dot plot. (**c**) Mean sample-level cell type proportions in acute rejection and non-rejection groups. (**d**) Violin plots comparing TSPO and LRBA expression across cell types in both groups.

We used GSE189536 single-cell transcriptomic data to examine TSPO and LRBA expression across cell populations in kidney allograft rejection. Following quality filtering, dimensionality reduction, clustering, and annotation, the final dataset contained 30,933 cells across ten major cell types (Figure 7a). Representative markers supporting these annotations are shown in Figure 7b.

We next compared cell composition in acute rejection (AR) and non-rejection samples. AR samples contained larger proportions of T cells, monocytes/macrophages, and NK cells, but smaller proportions of proximal tubular cells and endothelial cells (Figure 7c). These findings showed a shift in relative cell composition toward greater immune cell representation in rejection samples.

We then examined TSPO and LRBA expression across the annotated cell types. Both genes were detected in multiple immune and renal cell populations, including monocytes/macrophages (Figure 7d). Given their expression in this population and the larger relative proportion of monocytes/macrophages in AR samples, we selected these cells for exploratory virtual knockout analysis.

We used scTenifoldKnk to assess the potential effects of TSPO and LRBA loss in monocytes/macrophages. Following LRBA virtual knockout, the most affected genes included CEBPD, AC020916.1, EEF1A1, JUND, RPS15A, RPS3, RPL27, GLUL, HLA-DRB1, and UBA52 (Supplementary Figure S3a). Following TSPO virtual knockout, EEF1A1 was among the most affected genes, together with genes involved in antigen presentation: HLA-DRB5, HLA-DPA1, HLA-DPB1, HLA-DQB1, B2M, and HLA-DQA1 (Supplementary Figure S3b).

KEGG analysis linked genes affected by LRBA virtual knockout to ribosome, coronavirus disease, phagocytosis, and pathways involving allograft rejection, graft-versus-host disease, and the intestinal IgA immune network (Supplementary Figure S3c). Genes affected by TSPO virtual knockout were enriched in antigen processing and presentation, phagocytosis, and the intestinal IgA immune network, as well as pathways associated with allograft rejection, graft-versus-host disease, autoimmune thyroid disease, and viral myocarditis (Supplementary Figure S3d). These results suggested that TSPO and LRBA perturbation in monocytes/macrophages may affect antigen presentation, immune activation, and rejection-related pathways, supporting monocytes/macrophages as an important cellular context for the TSPO/LRBA signature in kidney transplant rejection. However, these computational predictions are hypothesis-generating and do not establish target-specific regulatory mechanisms.

### 2.7. Experimental Validation of TSPO and LRBA Expression in a Mouse Kidney Transplantation Model

**Figure 8:**
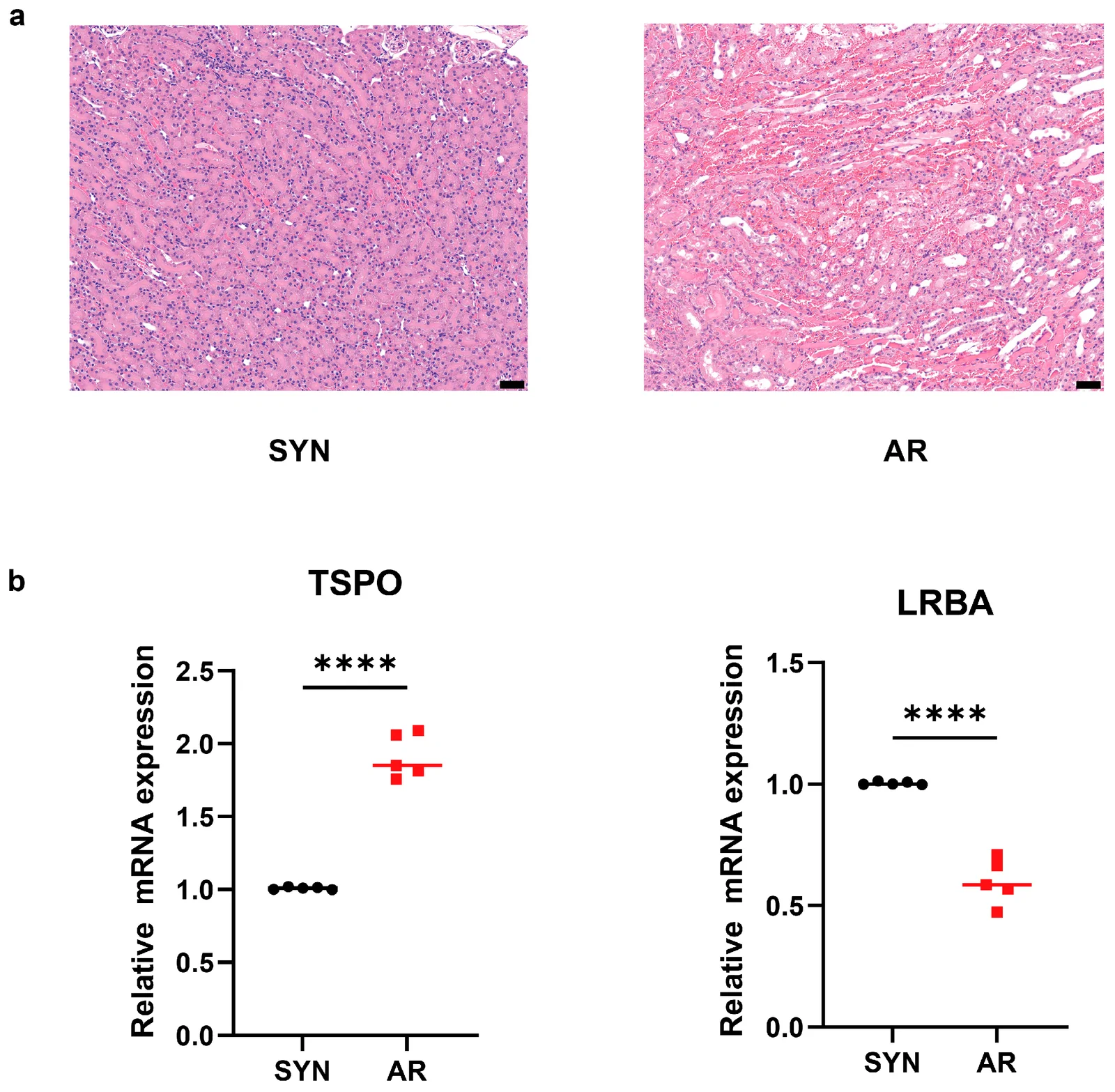
Experimental validation of TSPO and LRBA expression in mouse kidney transplantation model. (**a**) Representative hematoxylin and eosin (H&E) staining images of kidney grafts from SYN and AR groups on day 7 after transplantation. Scale bars, 50 μm. (**b**) Quantitative PCR analysis of TSPO and LRBA mRNA expression in mouse renal graft tissues on day 7 after transplantation. SYN, syngeneic transplantation; AR, acute rejection allograft. Values are shown as means with standard errors (SEM), with n = 5. **** *p* < 0.0001.

To further validate the expression patterns of TSPO and LRBA in vivo, we established a mouse kidney transplantation model according to a previously reported protocol and collected renal graft tissues on day 7 after transplantation. H&E staining showed increased inflammatory cell infiltration and tubular injury in the AR group, whereas renal tissue architecture was relatively preserved in the SYN group (Figure 8a). QPCR analysis showed that TSPO expression was significantly increased in the AR group compared with the SYN group, whereas LRBA expression was significantly decreased. (Figure 8b). These findings confirmed the opposite expression trends of TSPO and LRBA at the whole-graft level in an experimental model of acute renal allograft rejection and provided experimental support for their association with rejection-related immune activation.

### 2.8. Construction and Functional Analysis of the TSPO and LRBA Transcription Factor (TF) Network

To explore potential upstream regulation of TSPO and LRBA, we used NetworkAnalyst to predict transcription factors targeting the two genes. We identified 23 TFs for TSPO and 60 TFs for LRBA and constructed a TSPO/LRBA-TF regulatory network using Cytoscape (version 3.10.4) (Supplementary Figure S4a). Intersection analysis identified eight shared TFs: AR, HAND2, MITF, MYC, NFKB1, PHF8, POU5F1, and TBX5 (Supplementary Figure S4b).

We performed GO and KEGG enrichment analyses to examine the potential functions of the eight shared TFs targeting TSPO and LRBA. GO analysis showed that these TFs were mainly enriched in transcriptional regulatory functions. These included DNA-binding transcription activator activity, DNA-binding transcription factor binding, transcription coregulator binding, and regulation of DNA-templated transcription initiation. The TFs were also associated with miRNA metabolic and transcriptional regulation, organ formation, tissue remodeling, and cell differentiation-related biological processes (Supplementary Figure S4c). KEGG analysis showed enrichment in pathways related to cancer, myeloid leukemia, and osteoclast differentiation. Several inflammation- or immune-related pathways were also enriched, including NOD-like receptor signaling and cytosolic DNA-sensing pathways (Supplementary Figure S4d). These results suggested that the shared upstream TFs may mainly participate in transcriptional regulation and cell-state remodeling, with potential links to immune–inflammatory signaling in kidney transplant rejection.

We next compared the expression of the eight shared TFs between rejection and non-rejection samples. MYC and NFKB1 were significantly upregulated in rejection samples, whereas several other TFs, including AR, HAND2, MITF, and TBX5, were downregulated (Supplementary Figure S4e). These results suggested that MYC and NFKB1 may be closely associated with transcriptional changes during kidney transplant rejection. We then examined the correlations between the expression of these TFs and the hub genes. MYC and NFKB1 showed consistent positive correlations with TSPO expression and negative correlations with LRBA expression across several cohorts (Supplementary Figure S4f). Given the increase in TSPO and decrease in LRBA in rejection samples, these findings suggested that MYC and NFKB1 may be potential upstream regulators contributing to the TSPO-high/LRBA-low expression pattern during immune rejection.

### 2.9. Construction of Hub Gene–miRNA Network

To explore potential post-transcriptional regulation of TSPO and LRBA, we predicted miRNAs targeting the two hub genes using NetworkAnalyst based on miRTarBase v9.0. We identified 29 predicted miRNAs for TSPO and 204 for LRBA. Intersection analysis identified 20 shared miRNAs that may regulate both genes. We used these predicted interactions to construct a TSPO/LRBA-miRNA regulatory network in Cytoscape (version 3.10.4) (Supplementary Figure S5a). These findings provided a theoretical basis for potential miRNA-mediated regulation of TSPO and LRBA during kidney transplant rejection. Further experiments are needed to confirm these regulatory relationships.

### 2.10. Exploratory Molecular Docking of TSPO- and LRBA-Associated Compounds

We searched the GeneCards database for compounds associated with TSPO and LRBA. Molecular docking analysis showed favorable binding affinities between several candidate compounds and TSPO or LRBA. AJ2-30 had the strongest predicted binding affinity with LRBA, with a Vina score of -8.849 kcal/mol. ONO-2952 had the strongest predicted binding affinity with TSPO, with a Vina score of −8.581 kcal/mol, followed by midazolam, estazolam, emapunil, fludiazepam, diazepam, and oxazepam (Supplementary Figure S6a). Representative docking conformations showed that these compounds could fit into the predicted binding pockets of TSPO or LRBA (Supplementary Figure S6b). These exploratory docking results provide hypotheses for further experimental testing.

## 3. Discussion

TSPO and LRBA are mitophagy-associated genes that may be involved in immune regulation. TSPO is embedded in the outer membrane of mitochondria [34] and is also known as the peripheral benzodiazepine receptor [35,36,37]. But its precise physiological function is still unclear [38] and it has been linked to several immune processes. In a cardiac transplantation model, TSPO reduced antibody-mediated rejection by changing mitochondrial metabolism in B cells and suppressing B-cell activation [39]. TSPO has also been reported to limit inflammation through macrophage-related mechanisms in myocarditis [40]. LRBA shows widespread expression across human tissues and is associated with the Golgi apparatus and vesicular structures [41,42]. LRBA is closely linked to immune homeostasis. Biallelic LRBA mutations cause severe immunodeficiency and autoimmune disease [43,44], and LRBA deficiency has been reported to impair autophagy and promote antigen presentation and T-cell immunity [45]. LRBA may also negatively regulate antitumor immunity [46].These findings suggest that macrophage-related mitophagy genes may help characterize the immune features of renal allograft rejection.

In this study, we identified a TSPO-high/LRBA-low expression pattern associated with kidney allograft rejection across multiple renal biopsy and peripheral blood cohorts. The same expression directions were confirmed by qPCR in mouse renal grafts with acute rejection. This consistency between human transcriptomic data and the mouse model supports the association of increased TSPO and decreased LRBA expression with acute rejection. This pattern was linked to pathways involved in immunity and the extent of immune cell infiltration. The two-gene model showed modest discrimination of rejection and an association with graft survival. Single-cell analyses further supported monocytes/macrophages as an important cellular context for these findings. Together, these results connect TSPO and LRBA expression with the immune changes accompanying kidney allograft rejection.

Previous studies have developed transcriptomic signatures for kidney allograft rejection. The multicenter kSORT study evaluated a peripheral blood gene-expression assay for acute rejection [17], and Zhang et al. reported a blood gene expression signature for subclinical acute rejection [31]. Azad et al. identified an inflammatory macrophage-associated three-gene signature linked to allograft injury and graft survival [11]. Previous work also reported mitophagy-associated transcriptional changes in acute TCMR [26]. Building on these studies, we used mitophagy-focused screening to identify and characterize a TSPO-high/LRBA-low expression pattern associated with kidney allograft rejection. We examined this pattern across transcriptomic cohorts, explored its monocyte/macrophage context using single-cell data, and obtained additional support for the expression directions from mouse whole-graft qPCR. These findings add expression-based evidence for these candidates but do not establish macrophage-specific regulation or altered mitophagic activity. Our contribution is therefore exploratory biological characterization rather than a new machine learning method or an established clinical classifier.

Although TSPO and LRBA showed opposite expression changes, TSPO-high and LRBA-low samples were enriched in largely overlapping immune pathways. These involved antigen processing and presentation, signaling through cytokines, chemokines, and T-cell receptors, as well as NF-κB and TNF signaling. Given the correlation between TSPO and LRBA, this overlap may reflect shared expression variation and should not be considered independent evidence. Previous studies have reported that TSPO ligands suppress B-cell activation and reduce antibody-mediated graft rejection in an experimental heart transplant model [39]. TSPO has also been associated with macrophage-related inflammatory responses [40]. In contrast, LRBA is involved in endolysosomal homeostasis and autophagy [41], and LRBA deficiency has been associated with increased antigen presentation and changes in T-cell responses [45]. These observations provide a biological context for the associations found in our study. The enrichment of autophagy, mitophagy, phagocytosis, and ubiquitin-mediated proteolysis among our candidate genes further suggests that organelle quality control may be linked to the immune response in rejecting grafts.

The immune cell analyses suggest that the TSPO/LRBA pattern is related to changes in the graft immune environment. Higher TSPO expression was associated with greater proportions of γδ T cells and activated CD4 memory T cells. In contrast, higher LRBA expression was associated with smaller proportions of these cells and M0 macrophages. These associations are exploratory because CIBERSORT fractions are relative and compositional, which can influence both gene–fraction and fraction–fraction correlations. They do not establish changes in absolute cell abundance. The γδ T-cell estimates require tissue validation before subset-specific biological interpretation. Single-cell analysis showed that acute rejection samples contained larger proportions of T cells, monocytes/macrophages, and NK cells, but smaller proportions of proximal tubular and endothelial cells. These findings place the expression pattern in a graft environment with greater immune cell representation. They also suggest that changes in cellular composition may contribute to the differences observed in bulk tissue. Because both genes were expressed in several populations, their contribution to rejection may involve interactions among different immune and renal cell types. Higher TSPO expression may partly reflect increased myeloid-cell infiltration or graft injury, whereas lower LRBA expression may partly reflect a reduced contribution from parenchymal cells.

Single-cell analysis characterized TSPO and LRBA expression in monocytes/macrophages from kidney allograft samples. Virtual knockout analysis further linked the two genes to antigen presentation, phagocytosis, and allograft rejection pathways in this population. TSPO virtual knockout affected several antigen presentation genes, including B2M and the HLA genes DRB5, DPA1, DPB1, DQB1, DRB1, and DQA1. LRBA virtual knockout was also associated with immune-related pathways and ribosomal processes. These observations suggest possible roles for TSPO and LRBA in antigen presentation by monocytes/macrophages and their interactions with T cells during kidney allograft rejection. However, the virtual knockout predictions may be influenced by highly expressed genes and network composition, and several enriched KEGG terms may reflect a shared HLA-related signal. The virtual knockout results are therefore hypothesis-generating and do not establish specific regulatory roles for TSPO or LRBA.

The two-gene model showed modest discrimination, with AUCs of 0.680 in GSE98320 and 0.769 in GSE76882. However, adding LRBA did not significantly improve the AUC compared with TSPO alone. The incremental predictive value of LRBA therefore remains unproven, and TSPO alone may offer a more parsimonious alternative. These findings do not establish equivalence between the models or exclude a biological role for LRBA.

The signature was also associated with graft outcomes: patients with higher scores had poorer graft survival, and the AUCs for 1-, 2-, and 3-year outcomes were 0.712, 0.732, and 0.667, respectively. The association with both rejection status and subsequent graft survival suggests that the signature may reflect immune injury relevant to graft outcomes. Combining the signature with clinical and pathological indicators is a useful next step in assessing its clinical value.

The biopsy analyses primarily provide tissue-level evidence of a rejection-associated expression pattern. They do not establish additional diagnostic value beyond histology or a role in resolving borderline changes or equivocal biopsy findings. The association with graft survival is exploratory and does not establish prognostic value beyond clinical and pathological indicators. A potential future application is blood-based assessment of rejection-related immune activity, but the present blood findings are preliminary and do not support clinical implementation.

The peripheral blood analysis provided preliminary evidence of a similar TSPO-high/LRBA-low expression pattern to that observed in renal allograft tissue. However, this cohort included only 40 samples, and the wide confidence interval indicates uncertainty in the estimated discrimination. Differences between tissue and blood expression may also affect the performance of the tissue-derived model. These findings should therefore be considered exploratory and do not establish clinical utility for a blood-based assay. Further assessment in larger, independent blood cohorts is needed. In the mouse model, qPCR also confirmed increased TSPO and decreased LRBA expression in renal grafts with acute rejection.

Regulatory analyses identified MYC and NFKB1 as candidates associated with the TSPO-high/LRBA-low pattern. Both were increased in rejection samples. Across several cohorts, higher expression of these factors was associated with higher TSPO but lower LRBA expression. These consistent relationships suggest that MYC and NFKB1 may be involved in the transcriptional changes accompanying rejection. However, the potential involvement of the 20 shared miRNAs remains uncertain because miRNA expression and their regulatory effects on TSPO and LRBA were not examined in this study.

Molecular docking identified several compounds with favorable docking scores for TSPO or LRBA. ONO-2952 had the most favorable docking score for TSPO, whereas AJ2-30 had the most favorable score for LRBA among the compounds examined. Other TSPO-associated compounds included midazolam, estazolam, emapunil, fludiazepam, diazepam, and oxazepam. TSPO was originally called the peripheral benzodiazepine receptor [35,36]. Hauet et al. previously examined peripheral-type benzodiazepine receptor levels in a porcine renal autotransplantation model of ischemia–reperfusion injury [47]. However, that study did not establish the efficacy of benzodiazepines in preventing or treating acute allograft rejection.

The regulatory network, virtual knockout and molecular docking analyses are exploratory and hypothesis-generating. The predicted regulatory relationships and effects of virtual knockout require experimental validation. The docking analysis lacked redocking, decoy and reference-ligand controls, and TSPO was modeled as a monomer without a membrane environment. These limitations restrict the interpretation of the docking results. Docking scores do not confirm target binding, changes in gene expression, biological activity or therapeutic benefit, and cannot determine whether activation or inhibition of TSPO or LRBA would be beneficial in kidney transplant rejection. Effective doses and safety were not assessed. These findings therefore do not support the use of benzodiazepines for AR or establish that modulation of TSPO or LRBA can prevent or treat rejection.

Several limitations should be considered. Most analyses used public transcriptomic datasets, and differences in patient characteristics, rejection definitions, treatment regimens and platforms may have contributed to variation across cohorts. GSE21374 and GSE46474 also contributed to gene prioritization. Their reuse for survival and peripheral blood analyses may introduce selection bias and limits the independence of these findings. Further assessment in cohorts not used for gene prioritization is needed. The directions of the TSPO and LRBA expression differences were consistent across the sensitivity analyses. However, these findings do not exclude processing period effects, differences in cellular composition or other unmeasured factors. Rejection subtypes and control definitions differed across cohorts, and we did not evaluate TCMR and ABMR separately. The performance of the signature in each rejection subtype therefore remains uncertain. Incomplete information on biopsy indication, time after transplantation and immunosuppressive treatment also limited assessment of their potential effects on the findings. In addition, several datasets that we used originated from the same institutions, and GSE98320 included biopsies from 13 centers. Therefore, patient overlap across these datasets cannot be excluded and should be considered when interpreting the cross-cohort results. Larger prospective studies are needed before clinical diagnostic use can be considered. The peripheral blood analysis included only 40 samples and yielded an imprecise estimate of discrimination. The performance of the tissue-derived model in blood therefore requires further evaluation in larger, independent cohorts. The single-cell dataset included only nine rejection and two non-rejection biopsies, limiting reliable group-level inference. Individual cells within a biopsy do not represent independent biological replicates [48], and differential expression within macrophages was not assessed using biopsy-level pseudobulk analysis. These findings therefore do not establish macrophage-specific transcriptional regulation and require validation in larger, well-annotated single-cell cohorts. We did not perform dedicated comparisons with BK virus nephropathy, acute tubular necrosis, or calcineurin inhibitor toxicity. Therefore, the ability of the TSPO/LRBA signature to distinguish rejection from these alternative causes of graft injury remains unknown. The observed expression pattern may partly reflect inflammation or tissue injury shared across different graft pathologies. Future studies could include these pathological groups to assess rejection specificity and use analyses that account for cell composition to distinguish changes in cell abundance from changes in expression within individual cell types.

Although mouse qPCR supported the expression changes at the whole-graft level, protein levels and localization of TSPO and LRBA in graft macrophages were not examined. Immunofluorescence co-staining could help assess their localization, but this was not performed because of limitations in available experimental material. Further cell-specific and functional studies are needed to clarify their roles in rejection. Mitophagy, mitochondrial function, and mitophagic flux were also not directly measured. The designation of the candidate genes as mitophagy-associated reflects their annotation in the predefined gene sets and does not establish a mechanistic role in rejection. Functional studies are needed to determine whether the observed expression changes are accompanied by altered mitophagy.

## 4. Materials and Methods

### 4.1. Data Collection and Processing

We downloaded public transcriptomic datasets from the Gene Expression Omnibus (GEO). GSE25902 was used to find rejection-associated genes. GSE36059 and GSE50058 were used for model training and evaluation, and model selection was based on performance in both GSE36059 and GSE50058. GSE131179 was used for additional testing after model development. GSE129166 and GSE21374 were used to examine the expression of the ten genes in renal allograft biopsies, and GSE46474 was used to examine their expression in peripheral blood. GSE98320 and GSE76882 were used for two-gene model construction and evaluation, respectively. GSE21374 was used for graft survival analysis. GSE46474 was then used for peripheral-blood analysis. GSE189536 was used for single-cell analysis. GSE1563 was also included in the transcription factor analysis. Samples were grouped according to the deposited metadata.

The datasets used in this study were obtained from the Gene Expression Omnibus (GEO) database at the National Center for Biotechnology Information (https://www.ncbi.nlm.nih.gov/geo/ (accessed on 3 May 2026)). Each dataset was accessed through the GEO website using its GEO Series accession number. Rejection and non-rejection samples were identified based on the sample annotations in the corresponding GEO records, and the available gene expression data and associated sample information were downloaded. Group assignments followed the diagnostic annotations provided in the original datasets. The information of the datasets can be found in Supplementary Table S1.

Array features were matched to gene symbols using the annotation files for each platform. When several array features mapped to the same gene, we used the mean expression for analysis. Mitophagy-associated genes were obtained from seven human gene sets in MSigDB release 2026.1 which included 129 unique genes.

### 4.2. Differential Expression Analysis

GSE25902 contained 24 acute rejection samples and 96 non-rejection samples. We compared gene expression between the two groups using limma. Genes with |log2 fold change| > 0.5 and adjusted *p* value < 0.05 were considered differentially expressed.

Sensitivity analyses were performed to assess the effects of sample inclusion, normalization and adjustment for available covariates on the TSPO and LRBA expression differences in GSE25902. These analyses included exclusion of baseline biopsies, restriction of the control subset, adjustment for histological scores, restriction to samples processed on the same scanner, additional quantile normalization, and adjustment for cell composition proxies. Immune composition adjustment used the first two principal components of LM22 relative fractions and was compared with an unadjusted model in the same CIBERSORT-valid subset. All analyses were conducted at the biopsy level because verified patient-to-biopsy linkage was unavailable. Effect estimates, 95% confidence intervals and Benjamini–Hochberg-adjusted *p* values were reported.

### 4.3. Co-Expression Network Analysis

We used WGCNA to build a co-expression network from all 21,755 annotated genes in GSE25902. A power of 20 was selected because it was the lowest value at which the scale-free topology fit index reached 0.80. Modules were detected by dynamic tree cutting, with at least 30 genes per module and a merging threshold of 0.25.

We calculated Pearson correlations between module eigengenes and rejection status. Module membership represented the correlation of each gene with its module eigengene, while gene significance represented its correlation with rejection status.

### 4.4. Functional Enrichment and Protein Interaction Analysis

Gene Ontology (GO) and Kyoto Encyclopedia of Genes and Genomes (KEGG) enrichment analyses of the 32 candidate genes were performed using clusterProfiler (version 4.14.6). The background comprised all genes retained in the processed GSE25902 expression matrix that could be mapped to Entrez IDs using org. Hs.eg.db, yielding 19,213 unique Entrez IDs. For each analysis, background genes were further restricted to those represented in the corresponding annotation database. *p* values were adjusted using the Benjamini–Hochberg method, and adjusted *p* < 0.05 was considered significant.

Protein–protein interactions among the candidate genes were obtained from STRING. The organism was set to Homo sapiens. The STRING database version was 12.0.

### 4.5. Machine Learning and Model Interpretation

GSE36059 and GSE50058 were used for model training and evaluation. The two cohorts contained 411 and 101 samples, respectively. Penalized regression models were tuned by fivefold cross-validation with AUC as the tuning metric. Coefficients were extracted at lambda.min. We selected the model based on its AUC values in both the training and validation cohorts. After model development, all 113 models were additionally evaluated in GSE131179 using fixed model parameters, selected features, and training-derived preprocessing parameters. GSE131179 was used to explore the cross-platform generalizability of the trained model.

Model Combination and Evaluation: All machine-learning analyses were performed in R (version 4.4.1).Machine learning algorithms, including SVM, Ridge, glmBoost, Lasso, elastic net, logistic regression, stepwise glm, plsRglm, random forest (RF), GBM, and LDA, were used to construct 113 model configurations. Models were trained in GSE36059 and evaluated in GSE50058. ROC curves and AUC values were used to assess model performance. Performance in both cohorts informed model selection.

For the Ridge + Logistic pipeline, the 32 candidate genes were standardized using the means and standard deviations of the GSE36059 training cohort. Ridge-penalized logistic regression was used to rank the candidate genes. The penalty parameter was selected by fivefold cross-validation using AUC, and coefficients were extracted at lambda.min. Genes were ranked by the absolute values of their ridge coefficients, and the ten highest-ranked genes were retained. Coefficient-based ranking followed by selection of a fixed number of top-ranked candidates has also been used in previous research [49]. Because ridge regression does not inherently produce sparse models, the top-ten rule was an additional feature selection step. An unpenalized logistic regression model was then fitted using all ten selected genes to estimate their combined association with rejection and generate predicted probabilities. The fitted model and training-derived standardization parameters were applied to GSE50058, and discrimination was assessed using ROC curves and AUC.

For model interpretation, SHAP values were calculated for the fitted Ridge + Logistic model using the analytical form of SHAP for linear models. Gene expression values were standardized with the means and standard deviations from the GSE36059 training cohort. Under this standardized scale, each gene had a training-set background value of zero. Therefore, the SHAP value for each gene was calculated as the fitted logistic regression coefficient multiplied by the standardized expression value. Mean absolute SHAP values were used to rank gene contributions.

The expression of the ten model genes was compared in GSE129166, GSE21374 and GSE46474. Spearman correlation was used to examine the association between TSPO and LRBA within each cohort.

### 4.6. Two-Gene Model and Peripheral Blood Analysis

A logistic regression model was developed using TSPO and LRBA expression in GSE98320 (343 rejection and 274 non-rejection samples). Expression values were standardized using the means and standard deviations of the training cohort. The model was logit (*p*) = 0.259492 − 0.103523 × z_LRBA + 0.662075 × z_TSPO.

Logistic regression models for TSPO alone, LRBA alone, and their combination were fitted in GSE98320 and applied unchanged to GSE76882 using training-derived standardization. AUCs were reported with DeLong 95% confidence intervals and compared using paired, two-sided DeLong tests. Predicted probabilities were compared using the Wilcoxon rank-sum test.

GSE46474 included whole-blood samples from 20 rejection and 20 non-rejection patients. Gene expression was compared using exact Wilcoxon rank-sum tests. For exploratory blood assessment, the models were applied using GSE98320-derived standardization and coefficients, without further feature selection or refitting.

### 4.7. Graft Survival Analysis

We applied the fixed two-gene model to 282 biopsies in GSE21374. The median model score served as the cutoff for separating samples into two risk groups. Graft survival was plotted with Kaplan–Meier curves, and differences between groups were tested with the log-rank test.

### 4.8. Time-Dependent ROC Analysis

We used the R package timeROC to assess the predictive performance of gene expression for graft failure during follow-up, as previously described [50]. Follow-up time and graft failure status were treated as the survival outcome. Patients who experienced graft failure were considered events, whereas patients without graft failure were right-censored at their last recorded follow-up.

### 4.9. Gene Set Enrichment Analysis

We conducted GSEA separately for TSPO and LRBA in the 343 rejection samples from GSE98320. For each gene, its median expression served as the cutoff for defining high- and low-expression groups. We used limma to compare these groups and ranked genes by log2 fold changes. GO and KEGG gene sets were analyzed with clusterProfiler (version 4.14.6), including only sets with 10–500 genes. We applied the Benjamini–Hochberg method to adjust *p* values. Positive normalized enrichment scores indicated enrichment in the high-expression group, while negative scores indicated enrichment in the low-expression group.

### 4.10. Immune Infiltration Analysis

We estimated the relative proportions of 22 immune cell types in GSE76882 and GSE98320 using CIBERSORT and the LM22 signature matrix. For GSE76882, 153 biopsy samples, including 54 acute rejection samples and 99 normally functioning transplant controls, were analyzed using 1000 permutations and quantile normalization. We retained samples with CIBERSORT *p* < 0.05, leaving 71 samples from GSE76882 and 330 rejection samples from GSE98320. Spearman correlation was used to assess associations between TSPO or LRBA expression and estimated immune cell fractions and correlations among the immune cell fractions. Benjamini–Hochberg correction was applied within each gene across 22 cell types and across the 231 unique cell–cell pairs within each cohort.

### 4.11. Single-Cell RNA Sequencing Analysis

We used Seurat v5 to analyze the GSE189536 dataset. Libraries from the same biological biopsy were grouped together. The dataset included nine acute rejection biopsies and two non-rejection biopsies. Cells were retained if they had at least 100 and fewer than 6000 detected genes. Total RNA counts were required to be no more than 50,000. Mitochondrial RNA counts were required to be less than 25%. Predicted doublets were removed using a Seurat-compatible DoubletFinder procedure. The settings were pN = 0.25, pK = 0.09 and an expected doublet rate of 7.5%, with homotypic adjustment.

Expression was normalized using SCTransform v2. Harmony was used to correct biopsy-associated variation. We used 20 Harmony dimensions to cluster cells at a resolution of 0.1 and visualized them with uniform manifold approximation and projection (UMAP). FindAllMarkers was run with min.pct = 0.25 and logfc.threshold = 0.25 to identify marker genes. Cell types were annotated based on these markers. The final dataset contained 30,933 cells across ten cell types.

TSPO and LRBA expression was examined using the SCT assay. Cell-type proportions were calculated separately for each biopsy. The proportions were then averaged within each diagnostic group.

### 4.12. Virtual Knockout Analysis

Virtual knockout of TSPO and LRBA was performed separately in monocytes/macrophages using scTenifoldKnk. Each analysis used up to 3000 cells sampled across diagnostic groups. We selected 1500 variable genes and added predefined target and immune-related genes. Gene networks were reconstructed with nc_nNet = 5 and nc_nCells = 500. We compared the reconstructed and virtually perturbed networks to identify affected genes.

Genes with adjusted *p* < 0.05 were used for enrichment analysis. GO and KEGG enrichment analyses used Benjamini–Hochberg correction. The ten most affected genes were shown for each analysis.

### 4.13. Animals and Kidney Transplantation

Male C57BL/6 and BALB/c mice, aged 8 weeks and weighing approximately 24 g, were obtained from Beijing Vital River Laboratory Animal Technology Co., Ltd. and housed under specific pathogen-free conditions. All animal procedures were approved by the Tongji Hospital Laboratory Animal Welfare & Ethics Committee.

The transplantation procedure was adapted from a previously described mouse kidney transplantation protocol [51,52]. The syngeneic control group (SYN) received BALB/c to BALB/c kidney transplants, whereas the allogeneic group (AR) received C57BL/6 to BALB/c kidney transplants, with five recipient mice per group. Surgery was performed under anesthesia with sodium pentobarbital (65 mg/kg, intraperitoneal injection). Following a midline abdominal incision, the donor left kidney and its associated vessels were exposed. The kidney was flushed with histidine–tryptophan–ketoglutarate (HTK) solution and harvested with the renal artery, renal vein and ureter. The graft was preserved in ice-cold HTK solution before implantation.

After removal of the recipient’s left native kidney, the donor kidney was implanted below the level of the native renal vessels. Graft blood supply was restored by end-to-side anastomoses of the donor arterial segment and renal vein to the recipient abdominal aorta and inferior vena cava, respectively. Urinary drainage was established by direct anastomosis of the donor ureter to the recipient bladder.

Renal graft tissues were collected on postoperative day 7 for histological examination and RNA extraction. Tissue sections were stained with hematoxylin and eosin (H&E) to assess inflammatory infiltration and graft tissue injury. Tspo and Lrba expression was measured by quantitative real-time PCR. Each recipient contributed one renal graft sample and was treated as one biological replicate.

### 4.14. Quantitative Real-Time PCR

Total RNA was extracted from renal graft tissues using the Total RNA Rapid Extraction Kit (Fastagen, Shanghai, China). RNA was reverse transcribed using the high-capacity cDNA Reverse Transcription Kit (Applied Biosystems, Thermo Fisher Scientific, USA). Quantitative real-time PCR (qPCR) was performed using a SYBR Green kit (Vazyme). The transcript levels of the target genes were calculated as the ratio of their expression to that of Gapdh. Tspo and Lrba expression was normalized to Gapdh. For each sample, ΔCt was calculated by subtracting the Ct value of Gapdh from that of the target gene. The mean ΔCt of the SYN group was used as the calibrator, and ΔΔCt was calculated as ΔCt_sample − mean ΔCt_SYN. Relative expression was calculated using the 2^−ΔΔCt^ method. Each mouse contributed one graft sample and was treated as one biological replicate. The primers were as follows (Table 1):

### 4.15. Transcription Factor and miRNA Networks

Transcription factor (TF)–target associations for TSPO and LRBA were obtained from NetworkAnalyst. The underlying TF–target database was JASPAR. TFs associated with both genes were identified by intersecting the two lists. The network was visualized in Cytoscape (version 3.10.4). GO and KEGG enrichment analyses were performed for the eight shared TFs.

Shared TF expression was compared between rejection and non-rejection samples using limma. Correlations with TSPO and LRBA were assessed using Spearman correlation. These analyses used GSE25902, GSE36059, GSE50058, GSE1563 and GSE98320. Benjamini–Hochberg correction was applied within each cohort.

miRNA-target associations were obtained from NetworkAnalyst using miRTarBase v9.0. Shared miRNAs were identified by intersecting the TSPO- and LRBA-associated lists. The miRNA network was visualized in Cytoscape (version 3.10.4).

### 4.16. Drug Screening and Molecular Docking

Candidate compounds associated with TSPO and LRBA were retrieved from the GeneCards database (https://www.genecards.org/) and subsequently verified using PubChem (https://pubchem.ncbi.nlm.nih.gov/). Ligand structures were obtained from PubChem. The human TSPO model AF-P30536-F1-v6 from AlphaFold was used as the TSPO receptor. The LRBA receptor was the PH-BEACH domain structure from PDB 1T77, with chains A-D retained.

We prepared receptor files with AutoDockTools (version 1.5.7) and ligand files with Open Babel 2.3.2. AutoDock Vina 1.2.7 was used to dock the ligands to the receptors, and representative poses were displayed in PyMOL (version 3.1.6.1).

Blind docking was performed without specifying a predefined binding site. The search boxes covered the receptor structures with approximately 5 Å of padding on each side. For TSPO, the box center was (4.241, 4.199, −2.983) Å, with dimensions of 65.930 × 48.803 × 73.395 Å along the x, y and z axes, respectively. For the LRBA PH-BEACH domain structure, the box center was (18.388, 27.841, 52.620) Å, with dimensions of 90.069 × 116.661 × 172.415 Å. Receptors were kept rigid, the grid spacing was 0.375 Å, and exhaustiveness was set to 8.

### 4.17. Abbreviations

The abbreviations used in this study and their full terms are listed in Supplementary Table S2.

### 4.18. Statistical Analysis

Statistical analyses were performed in R 4.4.1. Differential expression analyses and the comparisons of the ten genes used limma. Correlation analyses used Spearman correlation, except for WGCNA module analyses, which used Pearson correlation. Benjamini–Hochberg correction was applied to the multiple tests specified above. *p* < 0.05 was considered significant.

Quantitative PCR data were presented as the mean ± standard error of the mean. The SYN and AR groups were compared using an unpaired two-tailed Student’s *t*-test in GraphPad Prism (version 10.3.1).

## 5. Conclusions

In this study, we identified a TSPO-high/LRBA-low expression pattern associated with acute kidney allograft rejection and immune activation. This pattern was observed across renal biopsy cohorts and supported by qPCR in mouse renal grafts, with similar trends in peripheral blood. Higher two-gene model scores were associated with poorer graft survival, while cellular analyses highlighted monocytes/macrophages and antigen presentation as relevant areas for further study. However, adding LRBA did not significantly improve diagnostic discrimination compared with TSPO alone. Together, these findings support further assessment of TSPO and LRBA as candidate biomarkers, including their potential use in blood-based assessment, and further study of their associations with immune responses in kidney transplant recipients.

## Figures and Tables

**Table 1:** Primer sequences used for quantitative real-time PCR.

Gene	Forward Primer	Reverse Primer
Tspo	GCCTACTTTGTACGTGGCGAG	ATGGCTGAATACAGTGTTGCC
Lrba	ATGGCTAGTGAAGACAATCGTG	GCCTCCTACCAGCAGGTTAAAG
Gapdh	AGGTCGGTGTGAACGGATTTG	TGTAGACCATGTAGTTGAGGTCA

## Data Availability

The public transcriptomic datasets analyzed in this study are available from the Gene Expression Omnibus (GEO) database under accession numbers GSE25902, GSE36059, GSE50058, GSE131179, GSE129166, GSE98320, GSE76882, GSE21374, GSE46474, GSE189536, and GSE1563. The experimental data generated in this study are available from the corresponding author upon reasonable request.

## Supplementary Materials

The following supporting information can be downloaded at: https://www.mdpi.com/article/10.3390/ijms27219830/s1.
