# Supplementary material for: TSPO and LRBA Expression Is Associated with Acute Renal Allograft Rejection: An Integrative Transcriptomic Analysis
Source: Int J Mol Sci. 2026 Nov 2;27(21):9830. doi: 10.3390/ijms27219830 (PMC13763261; doi:10.3390/ijms27219830)
Supplement: Supplementary file 1 [file ijms-27-09830-s001.zip › Supplementary Figures.pdf]

# Figure S1

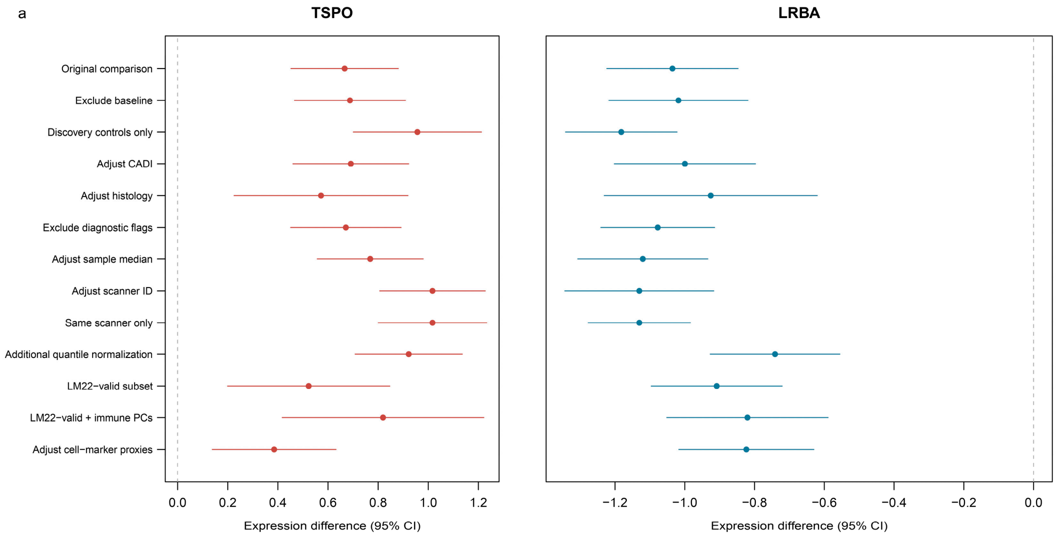


**Supplementary Figure S1.** Sensitivity analyses of TSPO and LRBA expression differences in GSE25902. Points show estimated log2 fold changes, and horizontal lines indicate 95% confidence intervals. Positive values indicate higher expression in rejection samples. Dashed lines indicate no difference.

Figure S2

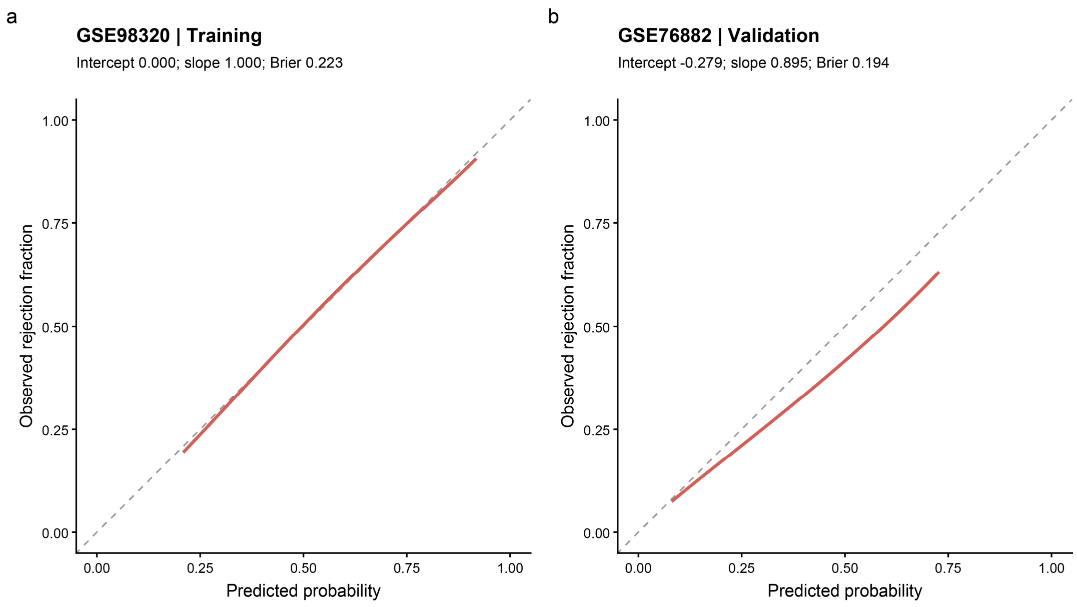


**Supplementary Figures S2**. **Calibration plots of the prediction model in the training and validation cohorts**. (a) GSE98320 training cohort . (b) GSE76882 validation cohort. The red lines show the relationship between predicted probabilities and observed rejection fractions. The dashed grey lines indicate perfect calibration. Calibration intercepts, slopes, and Brier scores are shown in each panel. The number of bootstrap resamples was 1,000.

# Figure S3

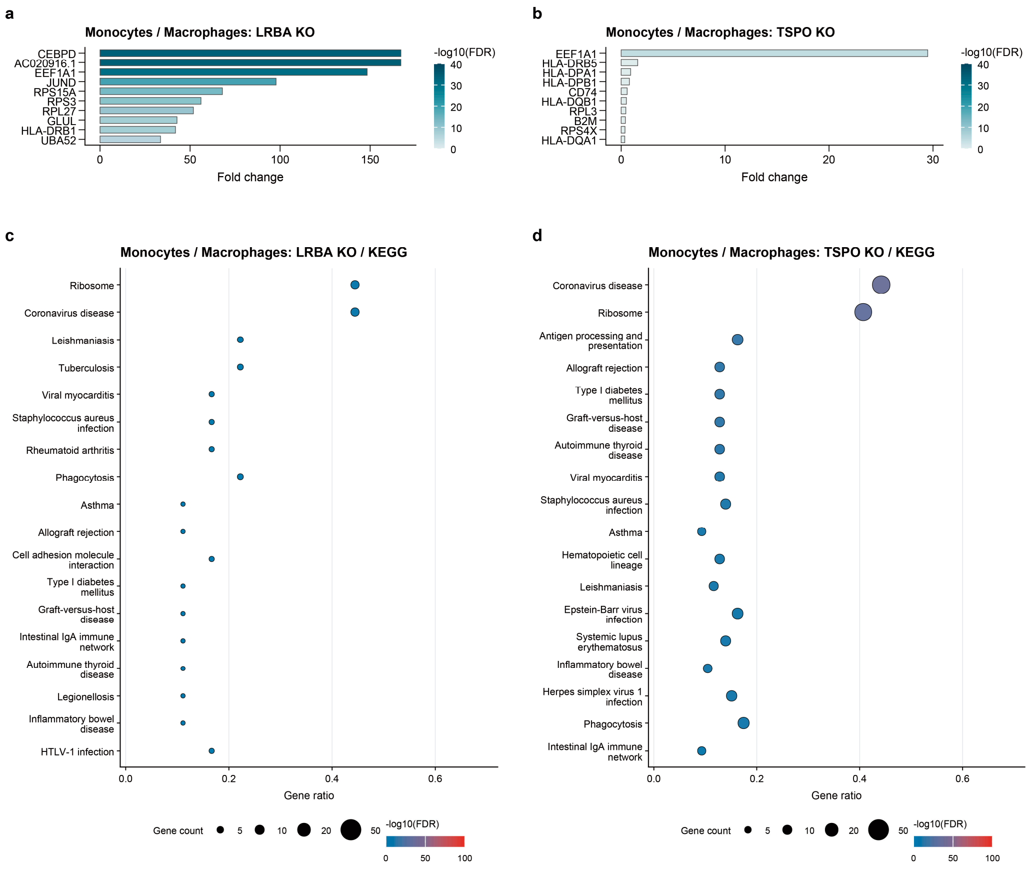


**Supplementary Figures S3. Virtual knockout analysis of LRBA and TSPO in monocytes/macrophages using scTenifoldKnk.** (a,b) The ten most affected genes following scTenifoldKnk virtual knockout of LRBA and TSPO, respectively, in monocytes/macrophages. (c,d) KEGG enrichment results for genes affected by LRBA and TSPO virtual knockout, respectively, in monocytes/macrophages.

Figure S4

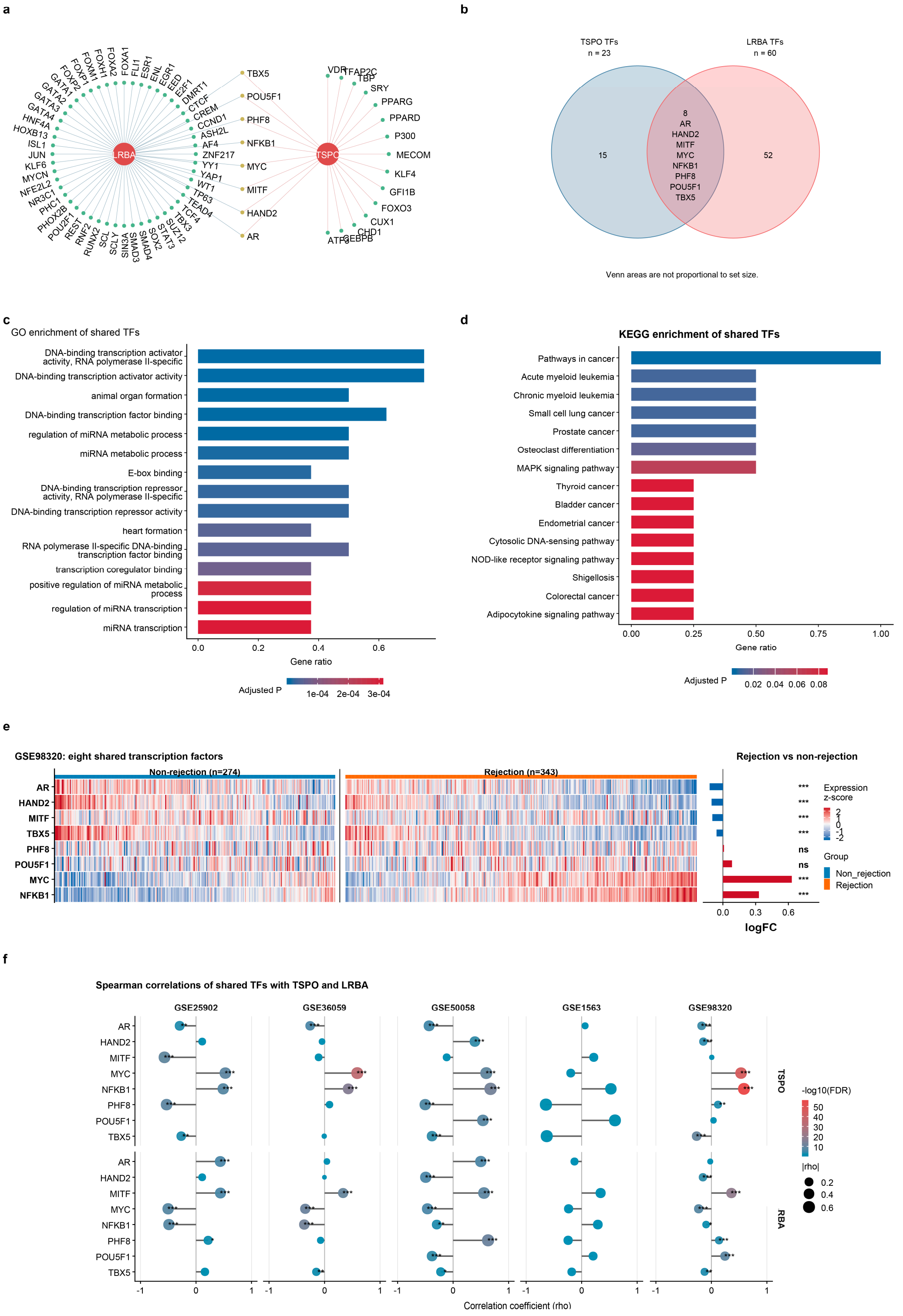


**Supplementary Figures S4. Construction and functional analysis of the TSPO and LRBA transcription factor (TF) network**. (a) Predicted links between transcription factors (TFs) and the target genes LRBA and TSPO. (b) Overlap of the TF lists for the

two genes, displayed in a Venn diagram. (c,d) Functional enrichment of the eight common TFs based on GO terms and KEGG pathways, respectively. (e) Heatmap of shared TF expression in GSE98320 rejection and non-rejection samples, together with differential expression summaries. (f) Associations of shared TF expression with TSPO and LRBA expression across cohorts. Expression differences were analysed with limma, and associations were evaluated by Spearman correlation. Within each cohort, P values underwent Benjamini–Hochberg correction.*adjusted $P < 0.05$; **adjusted $P < 0.01$; ***adjusted $P < 0.001$; ns, no significance.

# Figure S5

a

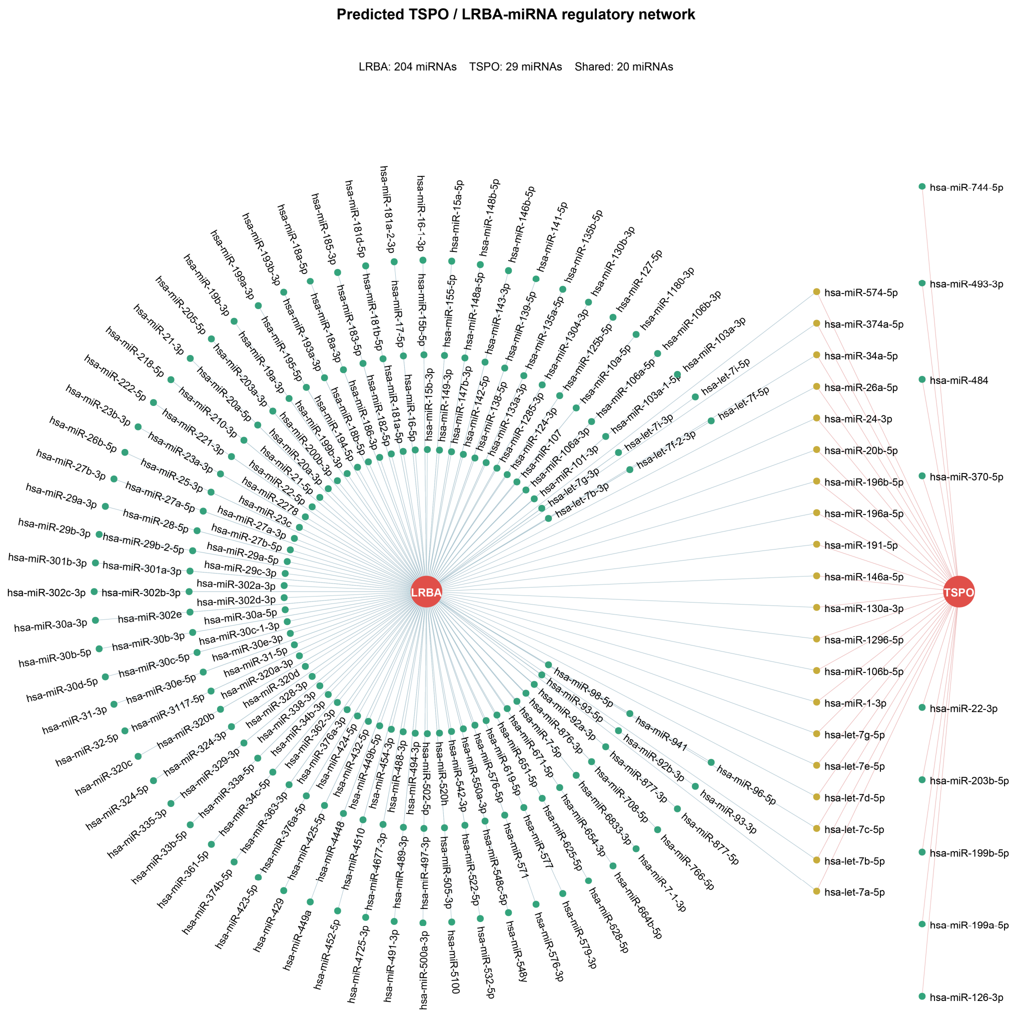


**Supplementary Figures S5. Construction of the hub gene-miRNA network.** (a) Network showing microRNAs (miRNAs) that may target LRBA and TSPO. Red nodes represent the two target genes, green nodes represent miRNAs associated with one

target, and gold nodes represent miRNAs shared by both targets. Edges show predicted miRNA-target associations.

Figure S6

**a**

| Gene | Gene source | Drug | PubChem CID | Vina score (kcal/mol) |
|---|---|---|---|---|
| LRBA | RCSB PDB 1T77 | AJ2-30 | 163902400 | -8.849 |
| LRBA | RCSB PDB 1T77 | 14C-Histidine | - | -5.598 |
| LRBA | RCSB PDB 1T77 | L-Histidine | 6274 | -5.44 |
| TSPO | AlphaFold AF-P30536-F1 | ONO-2952 | 11524127 | -8.581 |
| TSPO | AlphaFold AF-P30536-F1 | MIDAZOLAM | 4192 | -7.899 |
| TSPO | AlphaFold AF-P30536-F1 | ESTAZOLAM | 3261 | -7.283 |
| TSPO | AlphaFold AF-P30536-F1 | EMAPUNIL | 6433109 | -7.268 |
| TSPO | AlphaFold AF-P30536-F1 | FLUDIAZEPAM | 3369 | -7.155 |
| TSPO | AlphaFold AF-P30536-F1 | DIAZEPAM | 3016 | -7.086 |
| TSPO | AlphaFold AF-P30536-F1 | OXAZEPAM | 4616 | -7.019 |
| TSPO | AlphaFold AF-P30536-F1 | TRIAZOLAM | 5556 | -7.012 |
| TSPO | AlphaFold AF-P30536-F1 | LORAZEPAM | 3958 | -7.001 |
| TSPO | AlphaFold AF-P30536-F1 | FLUNITRAZEPAM | 3380 | -6.973 |
| TSPO | AlphaFold AF-P30536-F1 | HALAZEPAM | 31640 | -6.954 |

| Gene | Gene source | Drug | PubChem CID | Vina score (kcal/mol) |
|---|---|---|---|---|
| TSPO | AlphaFold AF-P30536-F1 | TEMAZEPAM | 5391 | -6.943 |
| TSPO | AlphaFold AF-P30536-F1 | CHLORDIAZEPOXIDE | 2712 | -6.862 |
| TSPO | AlphaFold AF-P30536-F1 | ESZOPICLONE | 969472 | -6.859 |
| TSPO | AlphaFold AF-P30536-F1 | CINOLAZEPAM | 3033621 | -6.83 |
| TSPO | AlphaFold AF-P30536-F1 | CLORAZEPIC ACID | 2809 | -6.664 |
| TSPO | AlphaFold AF-P30536-F1 | ALPRAZOLAM | 2118 | -6.66 |
| TSPO | AlphaFold AF-P30536-F1 | PRAZEPAM | 4890 | -6.535 |
| TSPO | AlphaFold AF-P30536-F1 | QUAZEPAM | 4999 | -6.521 |
| TSPO | AlphaFold AF-P30536-F1 | CLOTIAZEPAM | 2811 | -6.45 |
| TSPO | AlphaFold AF-P30536-F1 | ADINAZOLAM | 37632 | -6.326 |
| TSPO | AlphaFold AF-P30536-F1 | FLUMAZENIL | 3373 | -6.319 |
| TSPO | AlphaFold AF-P30536-F1 | ALPIDEM | 54897 | -5.893 |
| TSPO | AlphaFold AF-P30536-F1 | FLURAZEPAM | 3393 | -5.85 |
| TSPO | AlphaFold AF-P30536-F1 | CHLORMEZANONE | 2717 | -5.821 |

**b**

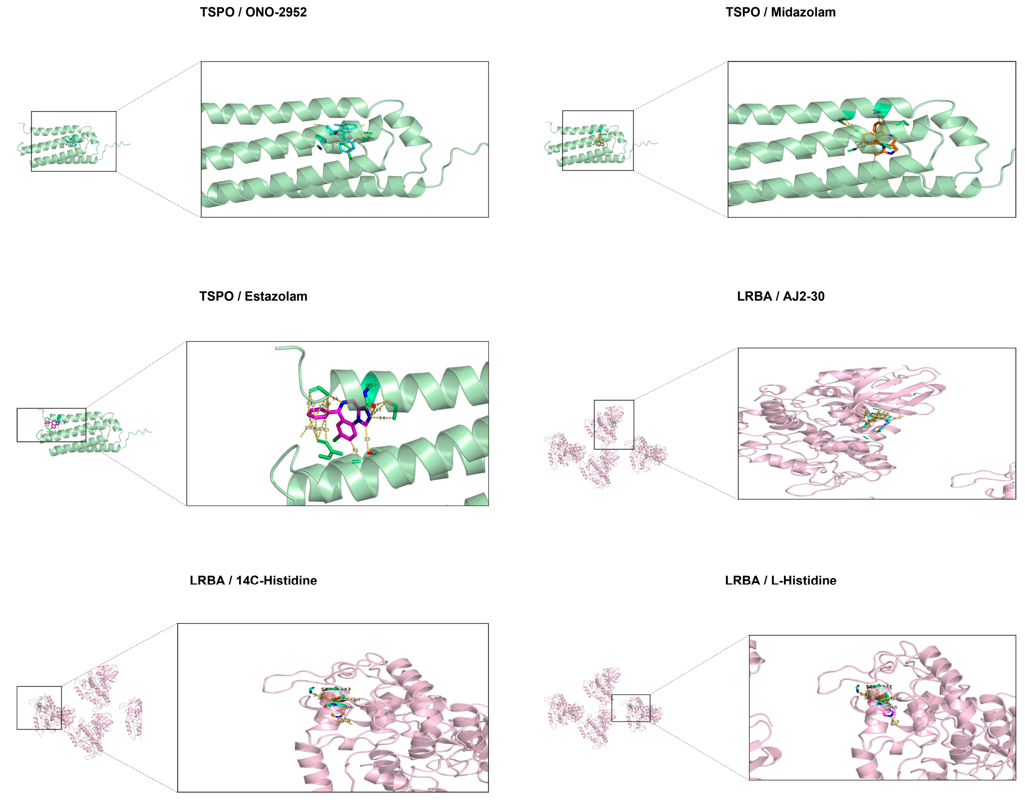


**Supplementary Figures S6. Exploratory molecular docking of compounds associated with TSPO and LRBA.** (a) Summary of candidate compounds, receptor structure sources, PubChem compound identifiers, and Vina docking scores. (b)

Representative docking poses showing TSPO with ONO-2952, midazolam, and estazolam, and LRBA with AJ2-30, 14C-histidine, and L-histidine.
